# Identification and Analysis of Co-Occurrence Networks with NetCutter

**DOI:** 10.1371/journal.pone.0003178

**Published:** 2008-09-10

**Authors:** Heiko Müller, Francesco Mancuso

**Affiliations:** 1 Department of Experimental Oncology, European Institute of Oncology, Milan, Italy; 2 Consortium for Genomic Technologies (Cogentech), Milan, Italy; University of Michigan, United States of America

## Abstract

**Background:**

Co-occurrence analysis is a technique often applied in text mining, comparative genomics, and promoter analysis. The methodologies and statistical models used to evaluate the significance of association between co-occurring entities are quite diverse, however.

**Methodology/Principal Findings:**

We present a general framework for co-occurrence analysis based on a bipartite graph representation of the data, a novel co-occurrence statistic, and software performing co-occurrence analysis as well as generation and analysis of co-occurrence networks. We show that the overall stringency of co-occurrence analysis depends critically on the choice of the null-model used to evaluate the significance of co-occurrence and find that random sampling from a complete permutation set of the bipartite graph permits co-occurrence analysis with optimal stringency. We show that the Poisson-binomial distribution is the most natural co-occurrence probability distribution when vertex degrees of the bipartite graph are variable, which is usually the case. Calculation of Poisson-binomial P-values is difficult, however. Therefore, we propose a fast bi-binomial approximation for calculation of P-values and show that this statistic is superior to other measures of association such as the Jaccard coefficient and the uncertainty coefficient. Furthermore, co-occurrence analysis of more than two entities can be performed using the same statistical model, which leads to increased signal-to-noise ratios, robustness towards noise, and the identification of implicit relationships between co-occurring entities. Using NetCutter, we identify a novel protein biosynthesis related set of genes that are frequently coordinately deregulated in human cancer related gene expression studies. NetCutter is available at http://bio.ifom-ieo-campus.it/NetCutter/).

**Conclusion:**

Our approach can be applied to any set of categorical data where co-occurrence analysis might reveal functional relationships such as clinical parameters associated with cancer subtypes or SNPs associated with disease phenotypes. The stringency of our approach is expected to offer an advantage in a variety of applications.

## Introduction

Biological research has experienced a paradigm shift in the last decade catalyzed by the availability of genome sequences and the resulting development of high-throughput technologies. The large data volumes produced by these novel technologies are often published as supplementary material and/or stored in extensive data repositories [1]. Functional interpretation of these data is an ongoing challenge. Co-occurrence analysis, based on the hypothesis that co-occurring entities are functionally linked, is a technique that has been used in three main areas of biological research:

1. Co-occurrence of genes in fully sequenced genomes.
2. Co-occurrence of words such as gene names, drug names, and keywords in titles, abstracts, or entire publications.
3. Co-occurrence of transcription factor binding motifs in sets of co-regulated genes.

Co-occurrence of genes in sequenced genomes relies on the fact that proteins do not function in isolation and are dependent on other proteins, either as direct binding partners, or as catalysts of substrates. Thus, when two proteins significantly co-occur in a large number of genomes or can be observed as fusion proteins in a subset of genomes, they are likely to be binding partners or enzymes needed for a specific metabolic pathway. Examples of those studies have been reported by [2]–[7].

Text mining is a quickly evolving field that aims at developing technologies helping to cope with the functional interpretation of large volumes of publications. Co-occurrence of gene names in publication abstracts, entire publications, or other gene-related databases has been used to derive co-occurrence networks with clear evidence that edges in those networks are reflecting functionally relevant relationships [8]–[11]. Gene names have also been analyzed for co-occurrence with other entities such as mutations [12], chemical compounds [13], and disease related keywords [14]. From the resulting networks, hypotheses about candidate genes involved in inherited diseases and drug targets can be derived. Clustering of gene related publications using keywords has been applied to enhance the quality of gene expression clusters [15], [16]. More general (non gene-centric) approaches try to organize the literature into functional areas based on co-occurrence of MeSH terms, keywords, diseases, phenotypes, chemicals, and similar objects of biomedical research interest [17]–[21].

Co-occurrence analysis of transcription factor binding motifs has been carried out in a variety of slightly differing ways in a wide range of organisms, including humans. [22]–[33]. The underlying hypothesis is that co-regulated genes, identified usually by gene expression studies, should contain specific combinations of transcription factor binding motifs in their upstream regulatory regions, the identification of which would allow the reverse-engineering of transcription regulatory networks [34].

We have recently applied co-occurrence analysis to studying published gene expression signatures and showed that co-occurrence patterns of genes reflect cancer signaling pathways [35]. Although co-occurrence analysis has a respectable history, the methodologies used in the studies mentioned above could not be easily applied to studying gene expression signatures. There are three main reasons that dictated the use of a different approach. First, gene expression signatures can vary in size by orders of magnitude. Obviously, the larger a signature the more likely it is to find two or more genes co-occurring in that signature. Thus, the significance of co-occurrences must be evaluated in the presence of considerable heterogeneity of co-occurrence probabilities among gene lists. As a consequence, the statistics used to evaluate the significance of co-occurrence events must reflect this heterogeneity. In particular, it must be based on list-specific co-occurrence probabilities. Second, in the vast majority of previous studies, co-occurrence is analyzed for pair-wise combinations of co-occurring entities. We found that the resulting stringency of this approach is not adequate for the analysis of published gene expression signatures [35]. Third, the null-model against which the significance of co-occurrences is tested does not work well for gene expression signatures. A common procedure is to use generic randomization of the entire data set under analysis or to select subsets of data entries randomly for comparison purposes. However, gene expression signatures are composed of distinct gene sets and the null-model must maintain this property, which is not guaranteed using these approaches. Furthermore, the list-specific nature of co-occurrence probabilities cannot be dealt with properly.

NetCutter was developed to address these challenges and to provide a generic tool for generating and analyzing co-occurrence networks. Although NetCutter has been developed for the analysis of gene expression signatures, it is based on abstract concepts that make it applicable to a wide variety of problems. The input is represented by a bipartite graph that is composed of list-entry pairs, which are stored in tab-separated text format. Co-occurrence of entries in lists is analyzed using pair-wise or higher order combinations of entries. The significance of co-occurrence is tested using a novel bi-binomial approximation of Poisson-binomial statistics (which is a binomial distribution with trial specific probabilities) that handles list-length-heterogeneity properly and provides a novel measure of association that is found to be superior to the Jaccard and the uncertainty coefficients. Occurrence probabilities are obtained from an edge-swapping procedure that maintains vertex degrees in the underlying bipartite graph and distinct sets of entries per list. As we shall see below, this procedure has a number of advantages over other possible null-models and permits co-occurrence analysis with near maximum stringency. Last but not least, NetCutter is equipped with a number of algorithms for the identification of network communities, vertex ranking, and convenience tools needed in the analysis of co-occurrence networks, or any undirected graph. We illustrate the utility of NetCutter in the identification of corresponding clusters of genes and publications from the PubLiME data set. PubLiME (Published Lists of Microarray Experiments) is a repository of published cancer related gene expression signatures (http://bio.ifom-ieo-campus.it/Publime). The concept of cluster correspondence follows from the bipartite graph representation of the data. Reversing the list-entry order in the bipartite graph permits identifying communities of entries as well as communities of lists. We show that communities of publications corresponding to communities of genes in the PubLiME data set can be used to generate hypotheses about the putative function of gene communities.

## Results

### The bipartite graph model of co-occurrence analysis

**Figure 1:**
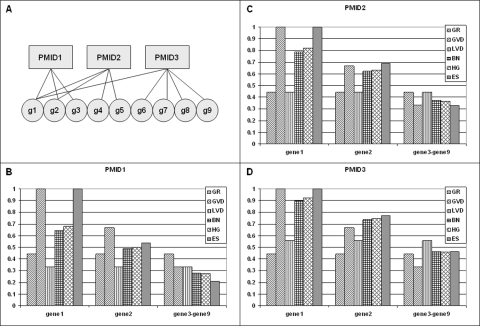
Bipartite graph data representation and null-models. A) PubMed IDs (PMIDx) and genes (gx) are represented by vertices of a bipartite graph. An edge indicates that a gene has been reported as differentially regulated in a specific publication. B–D) Occurrence probabilities of the bipartite graph shown in panel A as determined by six different null-models for PMID1 (B), PMID2 (C) and PMID3 (D): GR - generic randomization, GVD - gene vertex degree, LVD - list vertex degree, BN - binomial, HG - hypergeometric, ES - edge-swapping. See text for details of different null-models.

Co-occurrence analysis using NetCutter is based on the abstraction of list-entry pairs. Any entity that co-occurs with some other entity must be confined to some sort of container where co-occurrence is observed. For example, in the case of gene name co-occurrence in PubMed abstracts, the abstract is the container and the gene names are the co-occurring entities. Similarly, co-occurrence of transcription factor binding motifs is observed in gene promoters. The promoters are the containers where motif entities co-occur. The containers generally host more than one entity (otherwise co-occurrence would be impossible) and can be conveniently interpreted as lists. The co-occurring entities are the list entries. Lists and entries form a bipartite graph with one part of the graph representing lists and the other part representing entries. The presence of a given entry in a given list is indicated by an edge between the corresponding list and entry vertices. It is required that each entry can be linked to the same list only once. Without loss of generality, let's consider genes as entries and PubMedID_listIDs as lists in the following, unless otherwise specified (Fig. 1A). This interpretation of lists and entries has been applied in the co-occurrence analysis of published gene expression signatures [35].

### Occurrence probabilities and null-models

A prerequisite for co-occurrence analysis is the availability of occurrence probabilities of genes per list. The occurrence probabilities can be derived from randomizing the bipartite graph and are dependent on the choice of the null-model. A null-model creates an occurrence probability matrix where the occurrence probability for each list–gene pair is listed. As a general property of this matrix, the sum of all matrix elements must equal the number of edges in the bipartite graph. This is because each edge is linked to either side of the bipartite graph with certainty and therefore the sum of occurrence probabilities over all lists (which can be calculated as the row sum if genes are listed vertically or as the column sum if genes are listed horizontally) followed by summing the results over all genes must be 1 for every edge. The number of matrix elements is given by #genes*#lists and therefore the average occurrence probability for any null-model must be #edges/(#genes*#lists). As a consequence, different null-models will only be distinguished by the way they attribute occurrence probabilities to vertices with different vertex degrees but not by the average occurrence probability.

We consider six different strategies to randomize the bipartite graph. First, we could reconnect all edges of the graph randomly. The probability of being connected by an edge for a given list-gene pair is given by (1/#genes)*(1/#lists). Since there are #edges edges to be reconnected, the occurrence probability for a single list-gene pair is #edges/(#genes*#lists), i.e. equal to the average occurrence probability. Thus, this model provides equal occurrence probabilities for all gene-list pairs and does not consider vertex degrees. We call this model the generic randomization (GR) model in the following.

Second, we could disconnect the edges on only the list side of the bipartite graph and reconnect them randomly. The occurrence probability of a gene vertex would be given by (gene vertex degree)/#lists. The sum of these probabilities over all lists is equal to the gene vertex degree and the sum of all gene vertex degrees is equal to the total number of edges. Thus, the sum of all matrix elements is equal to the number of edges, as required. Since this model considers gene vertex degrees, we call it the gene vertex degree (GVD) model.

Third, we disconnect the edges on the gene side of the bipartite graph and reconnect them randomly. The probability of a list vertex being connected to a gene would be given by (list vertex degree)/#genes. The sum of these probabilities over all genes is equal to the list vertex degree and the sum of all list vertex degrees is equal to the total number of edges. Again, the sum of all matrix elements is equal to the total number of edges. Since this model considers list vertex degrees, we call it the list vertex degree (LVD) model.

In model four and five, we reconnect edges considering both gene and list vertex degrees and allow multiple edges between list-gene pairs. The occurrence probabilities in model four are calculated according to the binomial distribution. We calculate the probability of a list-gene pair for being connected as the cumulative binomial probability of the list-gene pair being chosen at least once in the process of randomly reconnecting the edges. This can be achieved by setting the number of trials equal to the gene vertex degree, the probability of success equal to the list vertex degree divided by the total number of edges, and the number of successes equal to 0. The occurrence probability of a list-gene pair is then given by the complement of this probability. This model is called the binomial (BN) model. In model five, we calculate occurrence probabilities according to the hypergeometric distribution. The number of successes in the sample is equal to 0, the sample size is equal to the gene vertex degree, the number of successes in the population is set to the list vertex degree, and the population size is the total number of edges. Again, the occurrence probability of a list-gene pair is obtained as the complement of this probability. We call this model the hypergeometric (HG) model. Calculating occurrence probabilities in this manner does not guarantee that the matrix elements add up to the total number of edges. Therefore, the matrices are normalized such that this condition is satisfied by multiplying each matrix element with the factor #edges/(observed matrix sum), which is generally quite close to 1, however.

In model six, we again consider vertex degrees, but we require that each list is composed of distinct sets of genes. Thus, multiple edges are forbidden. This condition is satisfied by applying an edge-swapping procedure during graph randomization. Edge-swapping works by randomly choosing two list-gene pairs from the bipartite graph and prior to performing the edge-swap, a test is performed to ensure that the two genes are not already linked to the respective target lists. This procedure is performed a large number of times. To ensure complete randomization of the graph, the number of swaps performed should be significantly larger than the number of edges. After performing *R* randomizations of the graph and counting the number of times a gene has been linked to a particular list, division of this number by *R* gives the occurrence probability of a gene in a given list. As will be shown below, edge-swapping produces occurrence probabilities that closely approximate occurrence probabilities obtained by generating a complete permutation set of the bipartite graph, counting the number of times a gene is found part of a list, and dividing this number by the total number of permutations. In the permutation model, the sum of occurrence probabilities of a gene over all lists equals the gene vertex degree (see below) and thus the sum of all matrix elements is the number of edges. Since permutation sets of bipartite graphs are difficult to calculate, we use the edge-swapping procedure as a close approximation and call this model the edge-swapping (ES) model.

Fig. 1 shows the occurrence probabilities of the different null-models for the bipartite graph shown in Fig. 1A. The GR model yields identical occurrence probabilities for all list-gene pairs, which is equal to the average occurrence probability in all models. In the other models, the occurrence probabilities deviate to varying extent from the average occurrence probability as a function of vertex degrees. In the GVD model, the deviations are a function of gene vertex degree and in the LVD model the deviations are dependent on list vertex degrees. In the remaining models, the deviations are functions of both the gene and the list vertex degrees. In all cases, larger than average occurrence probabilities are obtained for larger vertex degrees at the expense of smaller than average occurrence probabilities for smaller vertex degrees. From these data, it is difficult to choose the most effective null-model. A hint can be gleaned from gene1, however. Gene1 is present in all lists. Therefore, the co-occurrence probability of gene1 with other genes, which is calculated by multiplying the occurrence probabilities of gene1 and geneX for every list under study, should depend only on the occurrence probability of this other gene. In other words, the occurrence probability of gene1 in all lists should be 1.0. Only two models satisfy this constraint: The GVD and the ES models. Since the GVD model does not consider list vertex degrees, it seems that the ES model is the preferred null-model.

### Expected number of co-occurrences

As a general criterion for comparing the effectiveness of different null-models, we have to compare them for the number of expected co-occurrences. The most effective null-model will be the one that maximizes the expected number of co-occurrences. If the expected number of co-occurrences is larger, an observed number of co-occurrences in a real bipartite graph will be less significant and thus such a null-model permits co-occurrence analysis with higher stringency. The expected number of co-occurrences depends in an obvious fashion on the list vertex degree. If pair-wise co-occurrences are considered, the number of co-occurrences in a list of vertex degree N is given by the binomial coefficient N over 2. Larger lists will give rise to more co-occurrences and the number increases quickly with list vertex degree. The dependency of the expected number of co-occurrences on the gene vertex degree is less obvious and depends strongly on the null-model. A gene that is part of a list with vertex degree N will give rise to N-1 co-occurrences in that list. The null-model permits calculating the probability to find this gene in a given list. Thus, the expected number of co-occurrences of a gene is given by the sum of expected co-occurrences in all lists where for a single list the expected co-occurrences are given by (N_l_−1)*p_l_. N_l_ is the list vertex degree and p_l_ is the occurrence probability of the gene in that list as determined by the null-model.

**Figure 2:**
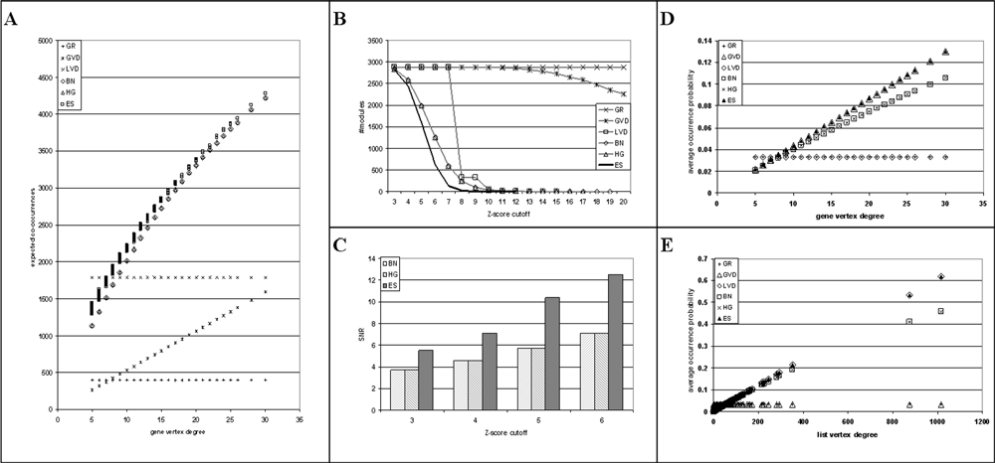
Properties of different null-models. GR - generic randomization, GVD - gene vertex degree, LVD - list vertex degree, BN - binomial, HG - hypergeometric, ES - edge-swapping. A) Expected number of co-occurrences in PubLiME data set: scatter plot of gene vertex degree against expected number of co-occurrences is shown. The expected number of co-occurrences of a gene is calculated as the sum of expected co-occurrences per list over all lists. The expected number of co-occurrences per list is given by list-vertex degree minus 1 times the occurrence probability of the gene in that list. B) Number of co-occurrence modules of size 3 present in at least 5 publications as a function of Poisson-binomial Z-score in the PubLiME data set. C) Signal-to-noise ratio (SNR) calculated as the number of modules in the real bipartite graph divided by the mean number of modules in 5 randomized bipartite graphs. D) Average occurrence probability of genes with the same vertex degree as a function of gene vertex degree. E) Average occurrence probability in lists with the same vertex degree as a function of list vertex degree.

We used the PubLiME data set [35] to calculate the expected number of co-occurrences with different null-models. The results are shown in Fig 2A. The expected number of co-occurrences was calculated for all genes in all lists using all null-models and the sum of expected co-occurrences per gene is shown as a scatter plot with the gene vertex degree on the x-axis and the expected number of co-occurrences on the y-axis. The results in Fig. 2A suggest the following ranking of null-models: GR<GVD<LVD<BN = HG<ES. The BN and the HG models perform in an essentially identical way. However, the ES model is the model that yields the largest estimates of expected co-occurrences. The results are also in line with the intuitive expectation that genes with higher vertex degree give rise to more co-occurrences. However, it can be seen that this is not true for all null-models. In particular, it is not true for the GR and the LVD models, which do not consider gene vertex degrees.

As outlined above, it is expected that the null-model that yields the highest estimates of expected co-occurrences should permit co-occurrence analysis with the highest stringency. In Fig. 2B, this hypothesis is tested directly again using the PubLiME data set [35]. For all null-models, co-occurrence analysis was carried out using module size 3 and support 5 (co-occurrence modules must be present in at least five publications). The choice of these parameters has been discussed in [35]. The number of co-occurrence modules was then determined that have a Z-score higher or equal than the cut-off shown in Fig. 2B. The Z-score is calculated from the mean and variance of the Poisson-binomial distribution as shown in the Materials and Methods section and published in [35]. More details on the probability distribution will be provided below. The GR and GVD models perform very poorly and identify large numbers of modules with high Z-scores. The LVD model performs a little better and approximates the BN and HG models at higher Z-score cut-offs. The BN and HG models give essentially identical results. However, the ES model is the model that yields the fewest number of significant co-occurrence modules and is thus the most stringent. The increased stringency of the ES model over the BN and HG models is also reflected in a higher signal-to-noise ratio calculated as the number of significant co-occurrence modules in the real bipartite graph divided by the number of modules found in a randomized bipartite graph (Fig. 2C).

The reason for the superior stringency of the ES model over all other models can be explained by examining the average occurrence probability per gene and list vertex degree. Fig. 2D and E show the average occurrence probability of genes with the same gene vertex degree as a function of the gene vertex degree. It can be seen that the ES model yields higher occurrence probability estimates for genes with higher vertex degrees as compared to the BN and HG models. In GR and LVD models, gene vertex degrees are ignored and occurrence probabilities for genes with large vertex degree are very small, which is compensated by larger occurrence probabilities for genes with small vertex degree. The GVD model is identical to the ES model in this setting. Fig. 2E shows the average occurrence probability of all lists with the same vertex degree as a function of list vertex degree. It can be seen that the ES model provides higher occurrence probability estimates for large lists as compared to the BN and HG models. In this setting, the LVD model performs like the ES model while the GR and GVD models yield small occurrence probabilities for large lists. Since it has been shown above that long lists and genes with high vertex degree are responsible for a large part of the total number of co-occurrences for the most stringent null-models, the null-model that provides larger occurrence probability estimates for genes and lists with high vertex degree at the expense of lower estimates for smaller degrees will be the most stringent because large occurrence probabilities make co-occurrence more likely and thus less significant. By these criteria, the ES model is the most stringent of all models tested.

### The ES model as an approximation of the permutation null-model

The data shown above have revealed that the ES model is the best of the models tested. One may wonder, however, whether yet more effective null-models can be found. An obvious choice would be the permutation model. In the permutation model, a complete permutation set of the bipartite graph is created such that each list is composed of distinct sets of genes. The number of graphs where a gene is present in a given list divided by the total number of permutations then provides the occurrence probability estimate. The permutation model is the ideal null-model because it is exhaustive. The problem is that a complete permutation set of bipartite graphs of some complexity is very time consuming to calculate. For example, the simple bipartite graph from Fig. 1A is part of a permutation set of 455 graphs. The number of permutations is increasing quickly as the numbers of genes and lists grow. However, since edge-swapping ensures that gene lists are composed of distinct sets of genes, each edge-swap produces a graph that is part of the permutation set of the bipartite graph. Edge-swapping can thus be viewed as a random sampling procedure from the permutation set of the bipartite graph. Therefore, occurrence probability estimates derived by edge-swapping should approximate those obtained from the permutation model.

**Figure 3:**
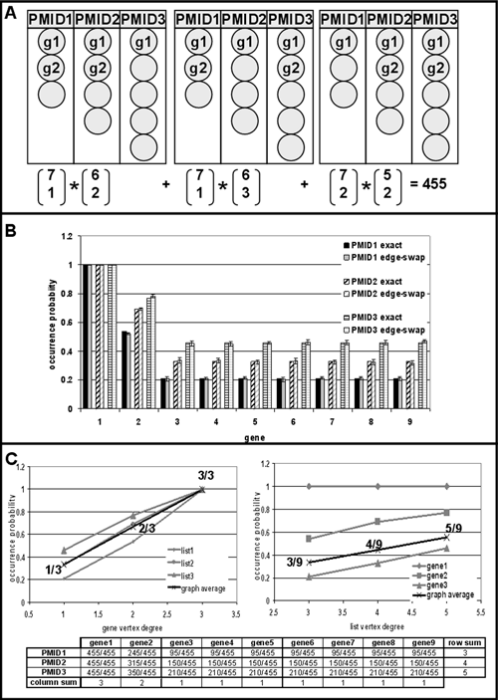
Edge-swapping as sampling from a complete bipartite graph permutation set. A) Calculation of the size of the permutation set of the graph shown in Fig. 1A. B) Precision of edge-swapping. Occurrence probability estimates are compared to their true values. See text for details. C) Individual and average occurrence probabilities are shown as a function of gene (left panel) and list (right panel) vertex degrees. The exact numbers of occurrences of each gene in each list are shown at the bottom and have been used to calculate exact occurrence probabilities. Note that row and column sums are adding up to vertex degrees. As a consequence, the average occurrence probability is a linear function of both gene and list vertex degrees.

We generated a complete permutation set of the graph shown in Fig. 1A to verify this hypothesis. The results are shown in Fig. 3. Fig. 3A shows how the number of possible permutations can be calculated. Gene1 is present in all lists and does not have an impact on the total number of permutations. Gene2, having vertex degree two, is present in two out of three lists in one out of three possible ways. The remaining genes have vertex degree 1 and can be freely chosen to fill the empty slots. We can now count exactly how many times a gene is linked to a list and divide these counts by 455, the size of the permutation set, to obtain exact occurrence probabilities. These numbers are shown in graphical form in Fig. 3B and in numerical form in Fig. 3C. Fig. 3B also shows the occurrence probability estimates obtained by edge-swapping side-by-side to the exact occurrence probabilities. The graph in Fig. 1A was subjected to edge-swapping 1000 times and the number of times a gene was found present in a list was divided by 1000 to obtain the occurrence probability. At each run, 100 random edge swaps were performed to ensure complete randomization of the graph. This procedure was repeated 10 times and the mean and standard deviation of occurrence probability estimates for each gene in each list are shown. In all cases, the mean differs from the real probability by less than two standard deviations, in most cases by less than one standard deviation. Thus, edge-swapping provides reliable estimates of exact occurrence probabilities as determined from a complete permutation set.

As an interesting observation, we provide evidence that occurrence probabilities are non-linear functions of vertex degrees in the edge-swapping model. This is illustrated in Fig. 3C. Individual and average occurrence probabilities are shown as a function of gene and list vertex degrees. Non-linearity of individual occurrence probabilities can be verified from the counts table underneath the plots. However, the average occurrence probability is found to depend on vertex degrees in a linear fashion instead. This is a consequence of the fact that occurrence probabilities of a gene over all lists add up to the gene vertex degree and that the occurrence probabilities of all genes for a given list add up to the list vertex degree. At the same time, since the most stringent permutation based null-model predicts non-linear dependencies of individual occurrence probabilities on vertex degrees, assuming such linearity in statistical models of co-occurrence will be linked to loss of stringency.

We conclude that the ES null-model is the null-model that permits co-occurrence analysis with the highest stringency among the models tested and that it closely approximates occurrence probabilities derived from an ideal permutation model. The increased stringency of the ES model over other models is a consequence of higher occurrence probabilities for genes and list with high vertex degrees, which are giving rise to a large part of all co-occurrences in the bipartite graph. Since large occurrence probabilities make co-occurrence more likely, the analysis becomes more stringent.

### Co-occurrence probabilities

Co-occurrence analysis can be thought of as a Bernoulli experiment with a binomial outcome (a given combination of entries is either present or not present in a given list). Thus, the Binomial distribution (BD) is a natural choice for judging the significance of the number of co-occurrences. However, the BD is defined for a probability of success which is equal in all trials. The list-specific nature of occurrence probabilities is not compatible with this condition (analysis of each list represents one trial), which means that co-occurrence analysis in the presence of list-length-heterogeneity is better described as a series of Poisson trials, where the probability of success varies from trial to trial. Therefore, the significance of co-occurrences must be evaluated using a binomial distribution with trial-specific probabilities, i.e. the Poisson-binomial distribution (PBD). The probability of success in a single Poisson trial can be calculated by multiplying the list-specific occurrence probabilities for the combination of genes under study. The number of occurrence probabilities that need to be multiplied is equal to the module size, i.e. the number of genes whose combination is studied. An observed number of co-occurrences for a combination of genes can then be evaluated using the PBD, which is given by the formula [36]:
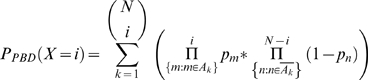
(18)


The structure of this formula is very similar to the structure of the formula used to calculate the binomial distribution, except that multiplication with a binomial coefficient is replaced by summation over individual terms, which makes calculation of P-values using (18) inefficient (note that equation numbering starts in the Materials and Methods section). Here, *A_k_* denotes the *k* ^th^ set of indices of the *i* lists where genes are co-occurring (“success”). There are 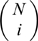 possible sets and summation is carried out accordingly. 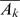 denotes the set of indices of *N−i* lists where genes are not co-occurring (“failure”). [36] have reported two fast procedures for calculating exact PBD P-values. However, both procedures work with probability ratios and suffer from numerical overflow/underflow problems for large numbers of trials. NetCutter uses two workarounds to circumvent this problem. One is based on using Poisson-binomial Z-scores, which can be calculated very easily instead (see below). The other relies on a fast approximation procedure for calculating Poisson-binomial P-values, which we call bi-binomial approximation (BBA) or bi-binomial distribution (BBD).

### Z-scores and P-values of BBD

Given the mean *μ* (1) and variance *σ^2^* (2) of PBD (see Materials and Methods), the Z-score associated with a given number of co-occurrences *x* is obtained as:
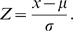
(19)


Considering the structure of formulae (1) and (2) (Materials and Methods section), PBD Z-scores can be calculated very easily and provide a simple estimate of the significance of co-occurrence modules. However, in contrast to normally distributed Z-scores, binomial and Poisson-binomial Z-scores do not correspond to the same P-value for different sets of probabilities of success. To see this, calculate for example the probability of success in a series of 100 Bernoulli trials with success probability 0.1 and 0.9 for the expectation of 10 and 90 successes, respectively. The Z-score will be 0 in both cases but the corresponding cumulative P-values are 0.5832 and 0.5487. Therefore, exact levels of significance cannot be derived from Z-scores alone. Thus, a fast and reliable procedure for calculating Poisson-binomial P-values is needed. The BBD approximation was developed to solve this problem.

The BBD approximation of PBD P-values follows from the relationship between the variance of PBD and the population variance of trial-specific probabilities of success. This relationship is shown in Materials and Methods to be described by (4):
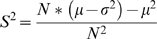
(4)


**Figure 4:**
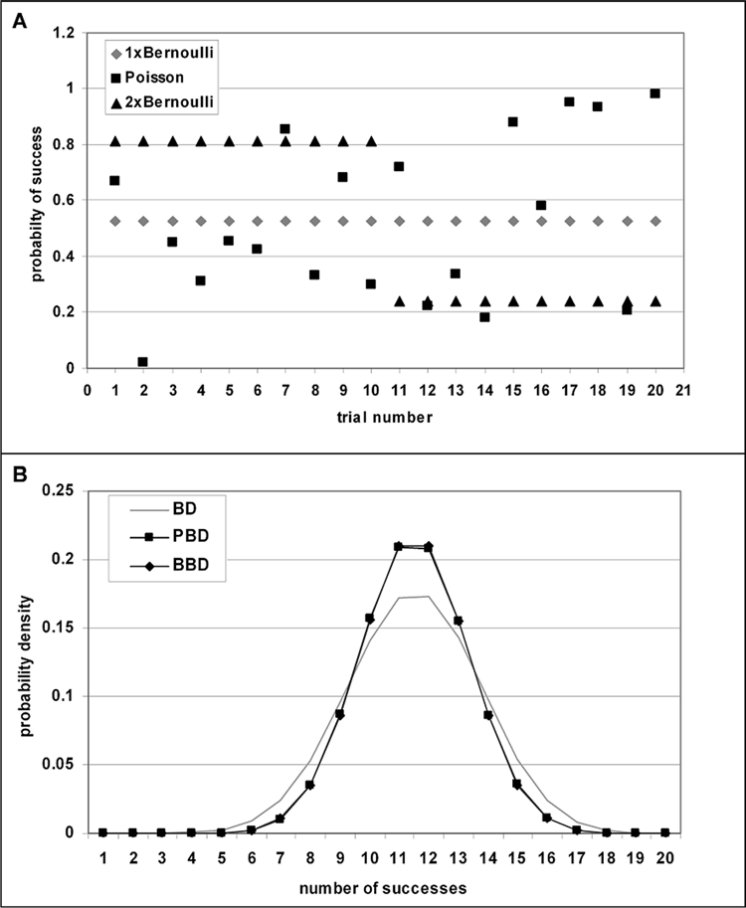
Bi-binomial approximation of Poisson-binomial distribution by replacing Poisson trials with two sets of Bernoulli trials. A) Three sets of 20 trials each with their respective probabilities of success are shown: Poisson trials (black squares), one set of Bernoulli trials with the average probability of success (grey diamonds), and the two sets of Bernoulli trials used to approximate the Poisson-binomial distribution (black triangles). B) The probability density functions corresponding to the trials in panel A are shown: BD - binomial distribution calculated from the average probability of success (grey line), PBD - Poisson-binomial distribution calculated from Poisson trials (black rectangles), BBD – bi-binomial distribution approximation calculated from the two sets of Bernoulli trials using the formula shown in Materials and Methods (black diamonds). Note that PBD is in general narrower than BD and that PBD and BBD are overlapping.

This equation shows that there is an inverse linear relationship between the population variance *S^2^* of the *N* trial probabilities and the variance of PBD *σ^2^*, which means that PBD becomes increasingly narrow as the variance of trial probabilities grows. It also shows that, for constant mean *μ* and number of trials *N*, the shape of PBD depends only on the variance of trial probabilities. Therefore, relationship (4) suggests an easy way to approximate PBD P-values. The P-value can be obtained by constructing a set of trial probabilities with equal variance as the original set of trial probabilities, which, however, are not all different. In other words, the series of Poisson trials can be replaced by two sets of Bernoulli trials with trial probabilities *p_1_* and *p_2_* constructed such that the variance is equal to the original set of trial probabilities. This strategy is illustrated in Fig. 4 and explains why this approximation is called bi-binomial. The details on how to obtain the values of the two sets of Bernoulli trial probabilities and the number of trials with *p_1_* and *p_2_* as probabilities of success are provided in the Materials and Methods section. The precision of the BBD approximation is discussed in supplementary material Simulation S1.

**Figure 5:**
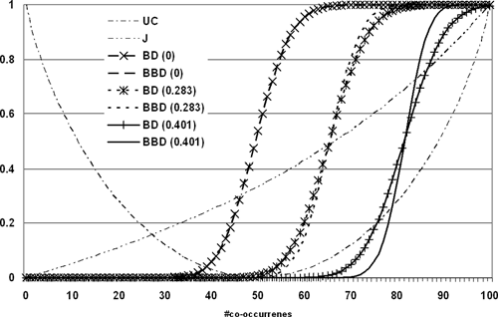
Comparison of measures of association. Two genes are assumed to occur in 100 out of 200 lists with occurrence probabilities that are constant (standard deviation 0), vary slightly (standard deviation 0.283) or strongly (standard deviation 0.401) from list to list. For each possible number of co-occurrences from 0 to 100, the uncertainty coefficient (UC), the Jaccard coeffcient (J), and the bi-binomial cumulative distribution function are calculated (BBD). For comparison purposes, the cumulative distribution function of the binomial distribution (BD) is calculated from the average co-occurrence probabilities, which are obtained by multiplying the occurrence probabilities of the two genes. Note that the expected number of co-occurrences depends on the variability in occurrence probabilities and that the same value of J and UC can be associated with positive, negative, or absence of association.

In order to evaluate whether BBD P-values as a significance measure of co-occurrence offer an advantage over other measures such as the Jaccard coefficient or the uncertainty coefficient, pair-wise co-occurrence of two genes in 200 lists with and without list-length-heterogeneity was studied (Fig. 5). Each gene is assumed to occur in 100 lists. Therefore, the occurrence probabilities of both genes over all 200 lists must add up to 100, regardless of list-length-heterogeneity. For simplicity, occurrence probabilities of both genes are assumed to be equal in any particular list. The co-occurrence probability in a list is then given by the square of the occurrence probability in that list. For all possible co-occurrences from 0 to 100, the Jaccard and uncertainty coefficients were calculated as detailed in the Materials and Methods section. In addition, cumulative BBD P-values were calculated using the co-occurrence probabilities as trial probabilities. To illustrate the advantage of BBD over BD as co-occurrence probability distribution, cumulative BD P-values of a BD with the same mean as BBD but constant trial probabilities is shown. These trial probabilities can be obtained by dividing the mean of BBD by the number of lists.

Three different cases of list-length-heterogeneity are considered in Fig. 5: No heterogeneity (standard deviation 0), heterogeneity with standard deviation 0.283 and heterogeneity with standard deviation 0.401 in the occurrence probabilities. The Jaccard and uncertainty coefficients are by definition insensitive to list-length-heterogeneity because differences in co-occurrence probabilities in a given list cannot be considered in their calculation. This is because both coefficients are defined by the counts of the four list classes: both genes absent, both genes present, first gene absent second gene present, and first gene present second gene absent, i.e. by the corresponding contingency table, which does not change with different list-length-heterogeneity. In the absence of list-length-heterogeneity, the cumulative P-values of BD and BBD (which are perfectly overlapping as expected) assume 0.5 at 50 co-occurrences, which corresponds to the expected number of co-occurrences calculated as (50 = 100 occurrences per gene/200 lists) ˆ2*200 lists. The uncertainty coefficient is found to be 0 and the Jaccard coefficient is 0.33333 at that point. When there is modest list-length-heterogeneity (standard deviation 0.283), the mean of BBD is shifting to the right. This is because the sum of squares of varying occurrence probabilities (i.e. the sum of co-occurrence probabilities used as trial probabilities, which is equal to the mean of BBD) is always larger than the sum of squares of constant occurrence probabilities with the same average occurrence probability (0.5). The corresponding BD in the presence of list-length-heterogeneity is obtained by dividing the expected number of co-occurrences by the total number of lists, which means assuming equal co-occurrences in all lists. This visualization is shown to illustrate how BBD (which is narrower than the corresponding BD) gives rise to a steeper cumulative distribution of P-values and as a consequence to more significant P-values for numbers of co-occurrence that are far from the expectation. As the level of list-length-heterogeneity grows (standard deviation of occurrence probabilities 0.401), the mean of BBD is shifted even further to the right and BBD P-values are distributed in a still steeper fashion as compared to corresponding BD P-values and the interval of non-significant co-occurrences is shrinking further. With modest list-length-heterogeneity, the expected number of co-occurrences is 66, which is associated with a Jaccard coefficient of 0.49 and an uncertainty coefficient of 0.075. In the case of large list-length-heterogeneity, the expected number of co-occurrences is 82 with J = 0.69 and UC = 0.32.

Taken together, these data show that the expected number of co-occurrences varies strongly with the level of list-length-heterogeneity and that the expected number of co-occurrences is associated with different values of UC and J. To complicate matters further, 66 co-occurrences (J = 0.49, UC = 0.075) represent significant positive association (P_BBD_ = 0.996) with equal list lengths, no significant association with modest differences in list length (P_BBD_ = 0.536) and strongly negative association (meaning one gene excludes the other) with strong list-length-heterogeneity (P_BBD_ = 0.00016). Thus, the same J and UC association measure is obtained for positive, negative, and absence of association. Therefore, the meaning of these measures cannot be interpreted properly in the absence of knowledge about the occurrence probabilities of the co-occurring entities. Furthermore, the data in Fig. 5 also show that neither J nor UC can distinguish between positive and negative association while this is easy with cumulative BBD P-values: Large P-values mean positive association and low P-values mean negative association. In summary, we conclude that BBD provides a novel association measure that offers a number of advantages over the existing contingency table based association measures Jaccard coefficient and uncertainty coefficient. The results in Fig. 5 also show that significance of association depends critically on the specific distribution of co-occurring entities over lists of varying length (because this distribution determines the occurrence probabilities) and that contingency table based methods (which cannot capture this distribution) should be avoided in the presence of significant list-length-heterogeneity.

### Generation of co-occurrence networks and the identification of communities

The procedures outlined above allow the identification of significant co-occurrence modules in any type of bipartite graph. Three user defined parameters have an impact on the stringency of co-occurrence analysis: The module size, the support, and the Z-score/P-value cutoff. The module size determines how many entries will be tested for co-occurrence, the support sets a lower boundary on the required number of co-occurrences, and the Z-score/P-value cutoff sets the significance threshold. In general, higher module size leads to more stringent co-occurrence analysis at the cost of computational complexity. The support parameter allows limiting this complexity by filtering out co-occurrence modules which co-occur less frequently than required by the support. The significance cutoff permits adjusting the signal-to-noise ratio, which is calculated as the number of co-occurrence modules observed in the real versus a randomized bipartite graph. The impact of these parameters on the stringency of co-occurrence analysis has been reported previously for the PubLiME data set [35] and is illustrated in a simulation study provided as supplementary material Simulation S1. From the set of significant co-occurrence modules, a co-occurrence network is generated by considering each entry a vertex and drawing an edge between any two vertices, which have been part of the same significant co-occurrence module [35].

An important question in the analysis of co-occurrence networks regards the presence of network communities. Communities can be understood as groups of vertices with the property that the number of edges running within groups is larger than expected by chance and that the number of edges running between groups is lower than expected by chance [37]. This problem of partitioning a graph is often referred to as the graph-cut problem (hence the name NetCutter). NetCutter is built on the Java Universal Network and Graph framework (JUNG) software package (http://jung.sourceforge.net), which provides algorithms for solving this problem. In particular, NetCutter implements the Bicomponent clustering algorithm [38], the Edge-Betweenness clustering algorithm [39], and the Exact Flow Community algorithm [40]. Furthermore, there is a clustering tool that is not part of the JUNG package, namely an algorithm identifying communities using eigenvectors of the modularity matrix [37]. The code for this algorithm was kindly provided by Mark Newman in C++ and ported to Java. In addition to these tools, NetCutter provides a number of convenience functions for the analysis of co-occurrence networks, such as testing the significance of lists reporting a set of entries making up a network community, ranking of vertices, random graph generators for topological analysis of co-occurrence networks, and others. Details on all functions are provided in the NetCutter software documentation.

One of the possible applications of NetCutter is illustrated below. This application is tightly linked to the bipartite graph representation of the data. Namely, NetCutter can be used to perform co-occurrence analysis of genes or list derived from the same bipartite graph. The network communities identified in each both reflect the same underlying structure of the bipartite graph. In the case of gene expression signatures stored in PubLiME, clusters of genes correspond to clusters of publications, which can reveal possible functions of gene clusters.

### Cluster correspondence and association studies

The co-occurrence analysis of the PubLiME data set published previously [35] identified 5 major network communities of genes with consistent functional annotations that are deregulated in cancer related gene expression signatures. This analysis was performed by considering all genes mentioned in a particular publication as a single signature, even though they might have been part of different tables and cluster analyses. Here we present an advanced analysis of the PubLiME data set where each table and/or cluster identified in a given publication is considered as a separate signature. This brings the total number of signatures to be analyzed to 1015 comprising a total of 7358 differentially regulated genes derived from 233 publications reporting cancer related signatures derived from human samples. We use this analysis to illustrate three major points: First, the set of communities reported previously is reproduced by this more fine-grained analysis. Second, the set of gene communities corresponds to a set of publication communities. Third, associations between publications and gene communities can be calculated with higher stringency using the edge-swapping null-model in conjunction with bi-binomial P-values as compared to binomial or hypergeometric statistics.

The bipartite graph to be analyzed is composed of PubMedID_listID-gene pairs (see supplementary material Table S1). Co-occurrence analysis was carried out in two ways: First, gene co-occurrence was analyzed and communities of co-occurring genes were defined by edge-betweenness clustering as described in Materials and Methods. Second, co-occurrence of PubMedID_listIDs was analyzed. To this end, the order of PubMedID_listID-gene pairs was reversed to form GENE-PUBMEDID_LISTID pairs. Thus, the lists in the resulting bipartite graph are formed by genes and the entries are the PubMedID_listIDs where the genes are reported as differentially regulated. Occurrence probabilities for the reversed bipartite graph can be obtained by transposing the occurrence probability matrix of the original bipartite graph. Since the gene communities identified in gene co-occurrence analysis reflect the structure inherent in the bipartite graph (which is not affected by reversing the list-entry order), co-occurrence analysis of the reversed bipartite graph will result in PubMedID_listID communities that reflect the same underlying structure in the bipartite graph. In other words, PubMedID_listID communities correspond to gene communities. In less abstract terms, the PubMedID_listID communities should correspond to sets of publications that report similar sets of genes as differentially regulated. The identification of communities of publications can help the researcher to easily identify publications studying genes in a gene community that is of interest to the researcher.

**Figure 6:**
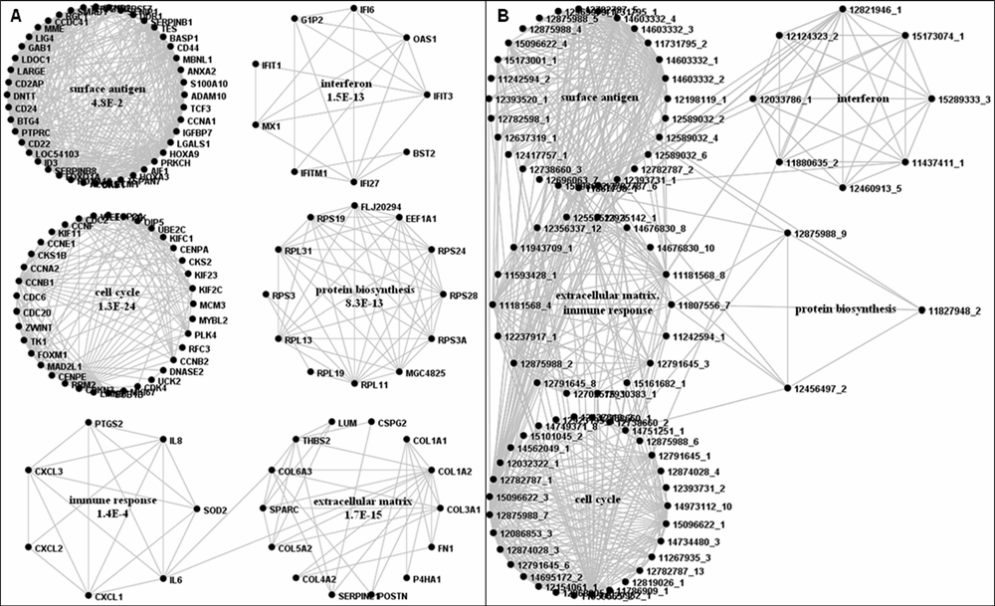
Gene and list communities in PubLiME. A) Co-occurrence analysis of the PubLiME data set was carried out as described in Materials and Methods. Gene communities in the co-occurrence network were identified by edge-betweenness clustering removing four edges corresponding to maximal graph modularity. Functional gene category enrichment analysis was carried out for community genes using the DAVID database. Benjamini corrected P-values are shown for the most significant categories. B) List co-occurrence analysis of the PubLiME data set was carried out on the bipartite graph with reversed list-gene order as described in Materials and Methods. List communities were identified by edge-betweenness clustering removing 130 edges corresponding to maximal graph modularity. Community names are derived from analyzing the probability of finding lists significantly enriched for genes in gene communities as part of the list community as described in Materials and Methods.

The results of both types of co-occurrence analysis are displayed in Fig. 6. Fig. 6A shows the gene clusters identified. The clusters are named after significant enrichments of gene categories as determined by functional category enrichment using DAVID [41]. The P-values shown are Benjamini corrected for multiple testing as reported by DAVID. The clusters are very similar to the clusters published previously [35]. There is one new cluster that is strongly enriched for ribosomal proteins (“protein biosynthesis” cluster), which has not reached significance in our previous analysis. The “surface antigen” cluster contains many genes that had been reported as part of the “signal transduction” cluster. Altogether, however, these results strongly support the notion that the gene clusters in the PubLiME data set can be reproduced by the more fine-grained analysis that considers sublists in each publication as separate signatures.

The corresponding clusters of PubMedID_listIDs are shown in Fig. 6B. There are five clusters, which have been named after their corresponding gene cluster. Only one cluster (the “extracellular matrix-immune response cluster”) cannot be separated by edge-betweenness clustering at the point of maximal graph modularity. To see that this naming is indeed justified, we needed to investigate how strongly a given PubMedID_listID is associated with a given gene cluster, i.e. how significant is the overlap of the genes reported in a gene cluster and the genes reported in a PubMedID_listID. Binomial or hypergemetric statistics are generally used to calculate this significance. However, the bipartite graph model in conjunction with the edge-swapping null-model offers a more fine-grained approach based on bi-binomial statistics.

**Figure 7:**
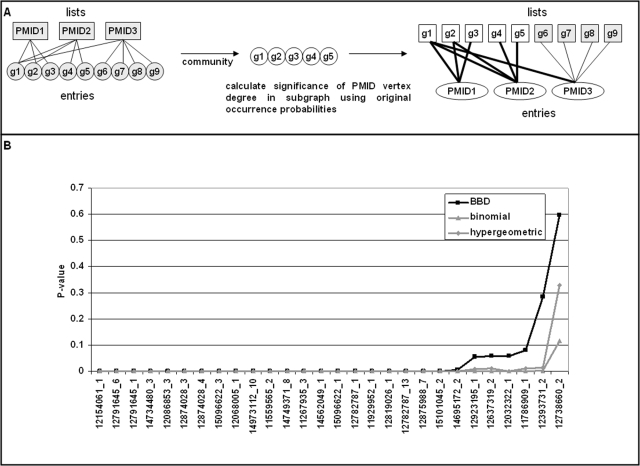
Lists associated with cell cycle cluster. A) Association is calculated as the cumulative bi-binomial probability of observing a given number of occurrences of an entry of interest in a subset of lists. When the subset of lists to be analyzed for association is derived from a community of entries, the underlying bipartite graph must be reversed such that entries become lists and vice versa. Occurrence probabilities for the transformed graph are obtained by transposing the occurrence probability matrix. B) The cumulative P-value of PubLiME publications reporting 37 genes of the cell cycle community (Figure 6A) is calculated using BBD, binomial and hypergeometric statistics. The lists are sorted by ascending P-value. BBD statistics are obtained following the scheme shown in panel A. Binomial and hypergeometric statistics are calculated as: Number of success: the number of cell cycle genes reported by the list. Number of trials: the number of genes reported in the cell cycle cluster. Probability of success: list length divided by 7358 total genes in the PubLliME data set used to generate the co-occurrence network.

The edge-swapping null-model determines occurrence probabilities in such a way that the number of genes in a given PubMedID_listID is associated with insignificant P-values in the context of the complete bipartite graph. However, when a subset of genes is analyzed, e.g. all the genes that are reported in a particular list, the P-value associated with the number of genes contained in this list will likely be highly significant according to how unlikely it is to obtain all the genes contained in a given list in a random draw from all genes present in the bipartite graph. Thus, PubMedID_listID association with a set of genes in the bipartite graph model can be calculated in the following way: The set of genes that is used to analyze association is used to extract a subgraph from the original bipartite graph where occurrence probabilities for each gene-PubMedID_listID pair are identical to those in the original bipartite graph (i.e. they are not recalculated by edge-swapping). The vertex degree of the PubMedID_listID vertices in the subgraph indicates the number of genes contained in each PubMedID_listID overlapping with the set of genes used to extract the subgraph. From the occurrence probabilities of the genes in a given PubMedID_listID, the bi-binomial P-value can then be calculated for every list vertex degree observed in the subgraph. In Fig. 7, the significance of association of the PubMedID_listIDs (see Fig. 6B) with the cell cycle cluster of genes (Fig. 6A) is calculated. For comparison, binomial and hypergeometric P-values are also shown. It can be seen that the bi-binomial P-value is larger than the binomial and hypergeometric P-values, which means that the strength of association is evaluated in a more stringent manner using BBD statistics (see Discussion for an explanation of this observation).

The analysis of significant associations between PubMedID_listIDs and gene clusters now permit answering the question whether there is correspondence between gene clusters and PubMedID_listID clusters. The naming of PubMedID_listID clusters shown in Fig. 6B is based on the number of PubMedID_listID that are significantly associated with gene clusters shown in Fig. 6A. First, for each gene cluster, all the PubMedID_listIDs that are associated with that cluster with more than 95% confidence (i.e. cumulative bi-binomial P-values> = 0.95) were identified. Second, the number of significant PubMedID_listIDs in each PubMedID_listID cluster was counted for every gene cluster. The significance of this number was then calculated using binomial statistics. The results of this analysis are shown in Table 1. Negative decadic logarithms of the binomial P-value are displayed. It is apparent that each PubMedID_listID cluster is strongly associated with at least one gene cluster, except for the “extracellular matrix-immune response” cluster, which is associated with two gene clusters. The strength of these associations suggests that the PubMedID_listID clusters are indeed corresponding to the gene clusters and that both the gene and the PubMedID_listID clusters reflect the structure of the bipartite graph representing the PubLiME data set.

**Table 1:** Cluster correspondence.

gene cluster\list cluster LC# (size)	LC1 (28)	LC2 (31)	LC3 (8)	LC4 (18)	LC5 (3)
**surface antigen (193 lists)**	**16 (4.52)**	1 (0.01)	0 (0.11)	1 (0.07)	0 (0.36)
**protein biosynthesis (15 lists)**	2 (2.13)	1 (1.12)	0 (0.95)	1 (1.55)	**3 (7.00)**
**interferon (64 lists)**	4 (1.50)	0 (0.06)	**8 (8.66)**	3 (1.57)	0 (0.76)
**cell cycle (122 lists)**	0 (0.01)	**22 (11.51)**	0 (0.21)	0 (0.05)	0 (0.52)
**extracelullar matrix (102 lists)**	1 (0.11)	1 (0.09)	0 (0.26)	**14 (9.36)**	0 (0.58)
**immune response (57 lists)**	2 (0.68)	2 (0.60)	1 (1.13)	**10 (8.32)**	0 (0.81)

For every list cluster (LC), the number of lists was determined that are significantly (P_BBD_> = 0.95) enriched for genes that are part of gene clusters. The significance of this number was evaluated using binomial statistics: #success – enriched lists in list cluster, #trials – number of lists reporting genes in gene cluster (e.g. 122 for cell cycle cluster), probability of success: size of list cluster divided by total number of lists (e.g. 31/1015 for LC2). The final P-value is obtained as 1 minus the cumulative binomial P-value. The negative decadic logarithm of the final P-value is shown in parentheses.

Details about all the lists analyzed are attached as supplementary material Table S2. Looking at these lists, some general conclusions about the gene clusters can be drawn. Cell cycle cluster genes have been found deregulated in a wide variety of tumor types such as colon cancer, breast cancer, in biliary tract cancer, pancreatic cancer, gastric cancer, prostate cancer, T-cell leukemia, glioma, acute lymphoblastic and myeloblastic leukemias, soft tissue sarcoma, neuroblastoma, as well as in a number of cellular model systems in response to different stimuli. Thus, the cell cycle cluster seems to consist of genes with a general role in oncogenesis. The surface antigen cluster instead seems to be derived preferentially from studies on leukemia. The interferon cluster genes are found deregulated in virus induced pathologies such as papilloma virus induced cervical cancer, and viral hepatitis. Immune response cluster genes were reported as differentially regulated in inflammatory conditions such as ulcerative colitis, Crohn's disease, and Helicobacter pylori infections. Genes of the extracellular matrix cluster seem to be associated with cancer progression studies and metastatic potential. For the protein biosynthesis cluster, there are 15 signatures that are significantly enriched for those genes. The cancers studied comprise medulloblastoma, glioblastoma, pancreatic cancer, soft tissue sarcoma, lung carcinoma, breast carcinoma, prostate carcinoma, multiple myeloma, and lymphocytic leukemia. The genes are also found deregulated in response to DNA damage. Although the number of signatures is limited, the variation in conditions where the genes are deregulated is compatible with the hypothesis that protein biosynthesis genes, as cell cycle genes, are deregulated in many cancer types, which might reflect the general property of cancer cells to divide and grow in an uncontrolled fashion.

## Discussion

Here we have investigated basic aspects of co-occurrence analysis and present a software tool, NetCutter, which can be used to identify and analyze generic co-occurrence networks. In NetCutter, a co-occurrence data set is represented as a bipartite graph with one part representing lists and the other part list entries whose co-occurrence patterns are studied. The bipartite graph representation of co-occurrence data sets allows the efficacy of different null-models to be tested systematically. We have shown that an edge-swapping procedure used to randomize the bipartite graph generates a null-model that allows co-occurrence analysis with the highest stringency. The other null-models tested here tend to underestimate occurrence probabilities of entries per list for lists and genes with high vertex degrees, i.e. for lists and genes where most co-occurrences are observed. As a result, co-occurrences are judged more significant than they really are.

Co-occurrence data sets with exactly equal lists lengths are likely to be the exception from the rule. It can be assumed that some list-length-heterogeneity will be present in most circumstances. An important consequence of list-length-heterogeneity regards the co-occurrence probability distribution used to evaluate the significance of the observed number of co-occurrences. Co-occurrence analysis in the presence of list-length-heterogeneity is best performed using the Poisson-binomial distribution (a binomial distribution with trial specific probabilities). However, calculating Poisson-binomial P-values for large numbers of lists is difficult using existing procedures [36]. We have presented an approximation to the Poisson-binomial distribution, called bi-binomial distribution, which is based on replacing the set of Poisson trials by two sets of Bernoulli trials. The resulting distribution reproduces the Poisson-binomial distribution nearly exactly and its P-values can be calculated with ease even for thousands of lists (see also supplementary material Simulation S1 for details on the precision of BBD). Importantly, BBD provides a novel measure of association, which is shown to be superior to existing measures such as the Jaccard coefficient and the uncertainty coefficient, whose values cannot be interpreted properly in the absence of knowledge about the occurrence probabilities of co-occurring entities.

It is worth noting that Poisson-binomial Z-scores are distinguished from Gaussian Z-scores by the fact that they do not correspond to the same P-value for different PBDs, BBDs, and even BDs. This is because the Z-score is an explicit part of the function defining the normal probability density while it is not part of the definitions of BD, PBD, and BBD densities. As a consequence, the simple Poisson-binomial Z-score based approach to evaluating significance of co-occurrence must be complemented with the BBD to approximate Poisson-binomial P-values in order to enable multiple testing corrections and to allow calculation of confidence levels in association studies precisely. However, NetCutter is equipped with a bipartite graph randomization tool that permits measuring the number of false positives due to multiple testing directly by comparing the number of significant co-occurrence modules in the real bipartite graph to the corresponding number in a randomized version thereof. Randomization is performed by edge-swapping in order to preserve vertex degrees. The resulting signal-to-noise ratios that are plotted for each Z-score/P-value cutoff provide a highly reliable and visually intuitive defense mechanism against false positives (see also supplementary material Simulation S1).

In the vast majority of co-occurrence studies, pair-wise co-occurrences have been analyzed using different statistical models. We have observed that the stringency of pair-wise co-occurrence analysis is far below the stringency of co-occurrence analysis using higher order combinations of co-occurring entities [35]. In NetCutter, co-occurrence analysis is preceded by occurrence analysis, i.e. the occurrence probability of each entry in each list is determined. Starting from occurrence probabilities, co-occurrence probabilities for any size of co-occurrence modules under study can be obtained by multiplying the respective list-specific occurrence probabilities. Given the list-specific co-occurrence probabilities, bi-binomial P-values are then calculated in exactly the same way for any module size. As a consequence, NetCutter can perform co-occurrence analysis for higher order combinations of co-occurring entries (i.e. larger module sizes) using the same statistical model. One of the benefits of using higher module sizes is robustness of the analyses in the presence of noise. This is because each edge in the resulting co-occurrence network is evaluated many times since every pair of co-occurring entries can be part of many higher order co-occurrence modules [35]. Another advantage is that implicit relationships between entries, which have never occurred together [18], can be derived as a natural by-product of using module sizes larger than 2. As shown in a simulation study (supplementary material Simulation S1), the result is a dramatic reduction of misclassifications at higher module sizes.

NetCutter can be used to calculate the strength of association between a subset of entries and lists reporting those entries. In this case, the analysis is performed on a subgraph of the original bipartite graph. The subgraph can correspond to communities of entries in the co-occurrence network, or any set of entries of interest. NetCutter will then calculate the significance of observing a given number of occurrences of an entry in the user defined subset of lists using bi-binomal statistics. This analysis mode corresponds to association studies with the advantage that the structure of the underlying bipartite graph (i.e. list length heterogeneity) is considered and handled appropriately using the bi-binomial distribution. As a consequence, association studies can be performed with higher stringency.

This result can be understood by examining the occurrence probability matrix that is implicitly assumed in performing binomial or hypergeometric tests for the significance of overlaps. In both tests, a gene is assumed to have an equal opportunity to be present in a list. Therefore, the probability of success for a gene to be part of a list is given by the list vertex degree divided by the total number of genes. In other words, both tests are implicitly based on the list vertex degree model, which has been shown previously to underestimate the occurrence probability and the expected number of co-occurrences for genes with high vertex degree (see Fig. 2A). Since the BBD P-values are calculated from the ES-model, which assigns higher occurrence probabilities to genes with higher vertex degree and more expected co-occurrences, the observed number of overlaps between a set of genes of interest and the content of a given list (which can be viewed as co-occurrence of the overlapping genes in that list) will be judged less significant when the overlapping genes are of high vertex degree (and vice versa when the overlapping genes are of low vertex degree) as compared to binomial or hypergeometric tests. Since the BBD P-values are derived from the most stringent ES null-model, BBD P-values provide a more reliable estimate for the significance of overlap.

Co-occurrence analysis of data represented as bipartite graphs permits visualizing the structure of the bipartite graph either as communities of list entries (genes) or as communities of lists (PubMedID_ListID) in co-occurrence networks. We have analyzed the PubLiME data set for the presence of corresponding gene and list clusters. In addition to previously published clusters of genes, we describe a novel gene cluster that is composed of protein biosynthesis associated genes [35]. We found that the corresponding clusters of PubMedID_ListID (gene expression signatures) are in general strongly enriched for genes reported in the corresponding gene cluster and that interrogation of corresponding clusters can be used to deduct hypotheses about the putative function of gene clusters.

In addition to co-occurrence analysis, NetCutter offers a number of tools for the analysis of co-occurrence networks, or any undirected graph. In particular, community identification is supported by four different community identification algorithms. NetCutter also offers a range of convenience functions that are of help in network analysis. Worthy of mentioning are the random graph generators that can provide control graphs for topological studies. The complete set of options is described in the software documentation.

In summary, we present a general framework for co-occurrence analysis with many potential applications. We illustrate a number of advantages of using the bipartite graph representation of data and the associated statistics. In particular, the identification of corresponding clusters permits the identification of functional subunits such as gene clusters on the one hand, and the generation of hypotheses about the function of those units by analyzing the corresponding list clusters on the other hand. Future developments will be directed towards the analysis of data sets that are considerably larger than the data sets analyzed so far. For example, co-occurrence analysis might be of interest for the analysis of single nucleotide polymorphism (SNP) data sets and association studies of genome variability with disease. Each patient is characterized by a specific range of SNPs. Co-occurrence patterns of patients according to their SNPs could be compared to clinical parameters with the aim of identifying genomic regions associated with disease. The increased stringency of association studies offered by NetCutter may be of use in the analysis of polygenic diseases where conventional methods fail. For being useful in this setting, NetCutter must be capable of analyzing bipartite graphs with millions instead of thousands of vertices.

## Materials and Methods

### Implementation of NetCutter

NetCutter is written in Java using NetBeans6 software (http://www.netbeans.info/) and tested on the Java Runtime environment 1.6.0.0. on a Windows XP Professional computer. The Java Runtime environment, which can be downloaded from http://java.sun.com/, must be installed on a computer that is intended to run NetCutter. NetCutter is provided as a single jar file and should run by double clicking the jar file, provided that the Java runtime environment is properly installed. NetCutter makes use of the following software packages and classes: JUNG version 1.3 (http://jung.sourceforge.net/download.html), Apache Jakarta Commons Collections 3.1 (http://jakarta.apache.org/commons/collections/), Cern Colt Scientific Library 1.2.0 (http://dsd.lbl.gov/hoschek/colt/), Xerces (http://xerces.apache.org/xerces2-j/index.html), Jama (http://math.nist.gov/javanumerics/jama/), Netlib Java LAPACK (http://www.netlib.org/lapack/), JFreeChart (http://www.jfree.org/jfreechart/), partition.java (http://astro.u-strasbg.fr/fmurtagh/mda-sw/java/partition.java).

### Bi-binomial approximation of Poisson-binomial distribution

The Poisson-binomial distribution (binomial distribution with trial specific probabilities) has recently been proposed as a statistic that properly handles largely differing sizes of gene expression signatures in meta-analysis of gene expression data [35]. Z-scores have been used to estimate the significance of co-occurrence because P-value calculation is cumbersome and error prone. Two methods reported by [36] suffer from numerical overflow/underflow problems when large numbers of Poisson trials with probabilities deviating significantly from 0.5 are being analyzed. Therefore, we propose a fast approximation of P-values based on a bi-binomial distribution. The bi-binomial distribution is a special case of the Poisson-binomial distribution where the probability of success can assume only two values. In order to achieve a good approximation of the underlying Poisson-binomial distribution, the values of these two probabilities and the number of trials where they are assumed must be determined carefully. As is shown in the following, the values of the two trial probabilities and their number of occurrences follow from the formula used to calculate the variance of the Poisson-binomial distribution and from the formula yielding the population variance of trial probabilities of the Poisson-binomial distribution to be approximated.

The mean *μ* and the variance *σ^2^* of the Poisson-binomial distribution are given by equation (1) and (2), respectively.
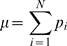
(1)

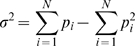
(2)
*p_i_* is the trial-specific probability of success and *N* is the total number of trials. For the sake of completeness, a formal proof of equation (1) is reported as supplementary material Proof S1 and the proof of equation (2) can be obtained in an analogous fashion.

The population variance *S^2^* of trial probabilities *p_i_* is given by equation (3).
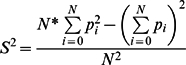
(3)


Rearranging equation (3) considering (1) and (2) leads to (4) and (5), where *p_a_* denotes the average trial probability of success and *q_a_* its complement.
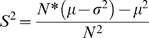
(4)

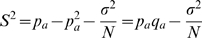
(5)Now let's define two trial probabilities *p_1_* and *p_2_*, which are used *N_1_* and *N_2_* times during the Poisson trials, respectively. Thus, *N_1_* and *N_2_* add up to *N*.
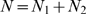
(6)


Considering (1), the average trial probability *p_a_* can then be obtained from (7).
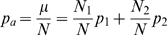
(7)


Using (7), *p_1_* can thus be calculated as (8).
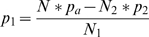
(8)


Similarly, considering (2), the variance *σ^2^* is given by (9).
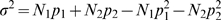
(9)


Substituting *p_1_* in (9) using (8) followed by substituting *σ^2^* in (5) by (9) leads to a quadratic equation for *p_2_* as a function of *p_a_*, *N*, and *S^2^*, as shown in equation (10).
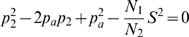
(10)


The solution to (10) is given by (11).
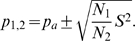
(11)


Setting *p_2_* to
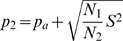
(12)
*p_1_* can be obtained from (8) and shown to be given by formula (13):
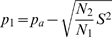
(13)Choosing *p_2_* as
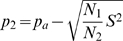
(12a)leads to *p_1_*

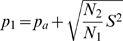
(13a)


Comparing (13a) to (12) and (12a) to (13), it can be seen that the formulae are identical except for the fact that *N1* and *N2* are reversed. Since the assignment of which set of trials is called *N1* and which set of trials is called *N2* is completely arbitrary, we can limit the remaining analysis on (12) and (13) without loss of generality.

Note that (12) and (13) do not guarantee that *p_1_* and *p_2_* are always confined between 0 and 1 for any combination of *N_1_* and *N_2_*. While probabilities smaller than 0 or bigger than 1 would still result in a distribution with the same overall variance as the original distribution, P-value calculation will be imprecise because the tails of the distribution will deviate significantly from the original distribution. Thus, we need to define the values *N_1_* and *N_2_* in such a way that *p_2_*< = 1 and *p_1_*> = 0. This can be achieved by evaluating (12) and (13).

Evaluating (12) for the condition that *p_2_*< = 1, solving the resulting inequality for *N_2_*, and considering (5), which relates *S^2^* and *σ^2^*, we obtain (14).
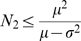
(14)


Similarly, evaluating (13) for the condition *p_1_*> = 0, solving the resulting inequality for *N_2_*, considering (5), which relates *S^2^* and *σ^2^*, and defining *μ_f_* the expected number of failures as N * (*1−p_a_*) (15),
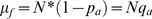
(15)we obtain (16)
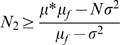
(16)


The meaning of these boundaries is best illustrated by considering a Poisson-binomial distribution whose variance is 0, i.e. that assumes 1 at *X* = *μ* and 0 otherwise. In this case (14) requires *N_2_*< = *μ* while (16) requires *N_2_*> = *μ*. These conditions can only be fulfilled contemporaneously when *N_2_* is set to *μ*. Intuitively, this means that there are *μ* trials with probability of success 1 and *N−μ* trials with probability of 0, resulting in a Poisson-binomial distribution with variance *σ^2^* = 0 and mean *μ*. When *σ^2^* is larger than 0, the choice of *N_1_* and *N_2_* is more flexible. However, since the choice of *N_2_* = *μ* is valid for all possible values of *σ^2^*, this is how NetCutter determines *N_1_* and *N_2_*. When *μ* is not an integer, *N_2_* is set to the integer closest to *μ*.

Having determined *p_2_* (12) and *p_1_* (13) as well a *N_1_* and *N_2_* (14, 16, 6), we can now calculate the bi-binomial approximation of the Poisson-binomial distribution in a fashion that is very similar to calculate the binomial P-value. With *q_1_* = 1−*p_1_* and *q_2_* = 1−*p_2_* we obtain:
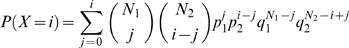
(17)


The summation is necessary because i successes can be obtained from any combination of *j p_1_* and *i−j p_2_* trials, where *j* can assume any value from 0 to *i*.

### Calculating Jaccard and uncertainty coefficients

For the purpose of comparing the efficacy of the bi-binomial distribution as a significance measure of co-occurrence, Jaccard and uncertainty coefficients (which are also called measures of association) were calculated using the formulae:
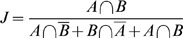



The Jaccard coefficient *J* is calculated as the number of times *A* and *B* occur together divided by the number of times *A* occurs without *B* plus the number of times *B* occurs without *A* plus the number of times *A* and *B* occur together [42].

The uncertainty coefficient [42] is calculated as:
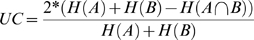


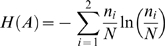


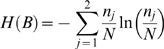


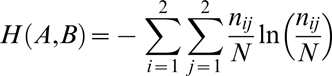

*H* is the entropy associated with *A*, *B*, and *AB*. For *A*, the entropy is calculated from the probabilities of *A* occurring in *n_1_* out of *N* lists (*n_1_/N*) and *A* not occurring in *n_2_* out of *N* lists (*n_2_/N*). Analogous calculations lead to the entropy associated with B. For *H*(*A*,*B*), the probabilities of *A* occurring without *B*, *B* occurring without *A*, *A* and *B* occurring together, and neither *A* nor *B* occurring in the lists are used.

### Co-occurrence analysis of the PubLiME data set

The bipartite graph to be analyzed is composed of 27619 PubMedID_listID-gene pairs (see supplementary material Table S1). Edge-swapping (1000 simulations, see above) was used to determine occurrence probabilities and gene co-occurrence was analyzed using module size 3 (co-occurrence of three genes), bi-binomial Z-score cutoff 6, bi-binomial P-value cutoff 1.0E-5, and support 5. Supplementary material Simulation S1 provides details on why module size 3 is chosen. The support parameter ensures that each 3-gene co-occurrence module is present in at least 5 signatures. We identified 1654 significant modules in the test data compared to 5 modules in a randomized bipartite graph, corresponding to a signal-to-noise ratio of 331. The co-occurrence network was generated from the significant co-occurrence modules by drawing an edge between each pair wise combination of genes that are part of the same co-occurrence module. Gene communities were identified in this network by edge-betweenness clustering removing 4 edges, which resulted in a maximal network modularity of 0.63. Modularity is calculated as described by [43].

For the identification of PubMedID_listID clusters, the PubMedID_listID-gene pairs in the original bipartite graph were reversed to form gene-PubMedID_listID pairs. Occurrence probabilities were obtained by transposing the original occurrence probability matrix determined by edge-swapping as described above. PubMedID_listID co-occurrence was analyzed using module size 5, Z-score cutoff 6, bi-binomial P-value cutoff 1.0E-5, and support 3. Please note that the choice of these parameters is dictated by the parameters used in gene co-occurrence analysis. The reversal of the bipartite graph necessitates the support parameter used in gene co-occurrence analysis (5) to be used as module size in PubMedID_listID co-occurrence analysis and the module size used in gene co-occurrence analysis (3) to be used as the support parameter in PubMedID_listID co-occurrence analysis if the scope of the analysis is the identification of PubMedID_listID clusters that correspond to gene clusters identified before. The significance cutoffs remain unchanged. PubMedID_listID co-occurrence analysis revealed 448 significant co-occurrence modules in the real bipartite graph and 6 significant co-occurrence modules in the randomized bipartite graph with a signal-to-noise ratio of 75. Communities in the resulting co-occurrence network were identified by edge-betweenness clustering removing 130 edges. The resulting maximal network modularity was found to be 0.47.

## Supporting Information

Proof S1 Proof of Poisson-binomial mean.
(0.03 MB PDF)

Table S1 PubLiME dataset.
(1.49 MB XLS)

Table S2 Cluster correspondence.
(0.33 MB XLS)

Simulation S1 (0.42 MB PDF)
