# Supplementary material for: Identification and Analysis of Co-Occurrence Networks with NetCutter
Source: PLoS One. 2008 Sep 10;3(9):e3178. doi: 10.1371/journal.pone.0003178 (PMC2526157; doi:10.1371/journal.pone.0003178)
Supplement: Proof S1 — Proof of Poisson-binomial mean. (0.03 MB PDF) [file pone.0003178.s001.pdf]

Mean and variance of Poisson-binomial distribution (PBD)

The PBD is given by:

$$P_{PBD}(X = i) = \sum_{k=1}^{\binom{N}{i}} \left(\prod_{\{m:m\in A_k\}}^{i} p_m * \prod_{\{n:n\in \overline{A_k}\}}^{N-i} (1 - p_n) \right) \quad (1)$$

Here, p_m and p_n indicate the probability of success (i.e. the co-occurrence probability of genes obtained by multiplying their occurrence probabilities in the respective lists) in the mth and n^{th} list with m <> n. A_k denotes the k-th set of indices of the i lists where genes are co-occurring. There are $\binom{N}{i}$ possible sets and summation is carried out accordingly. $\overline{A_k}$ denotes the set of indices of N-i lists where genes are not co-occurring.

The Mean μ of PBD is calculated by multiplying the number of successes by their respective probabilities and summation over all possible successes from 1 to N where N is the number of lists. If the co-occurrence probability in the *i-th* list is designated by p_i, this calculation evaluates to:

$$\mu = \sum_{i=1}^{N} p_i \quad (2)$$

Thus, the expected number of co-occurrences is equal to the sum of co-occurrence probabilities over all lists. To prove (2), we multiply out (1) and rearrange all terms such that all product terms of the same length are shown in the same column (Fig. Proof_S1).

Fig. Proof_S1

Calculating the mean of PBD

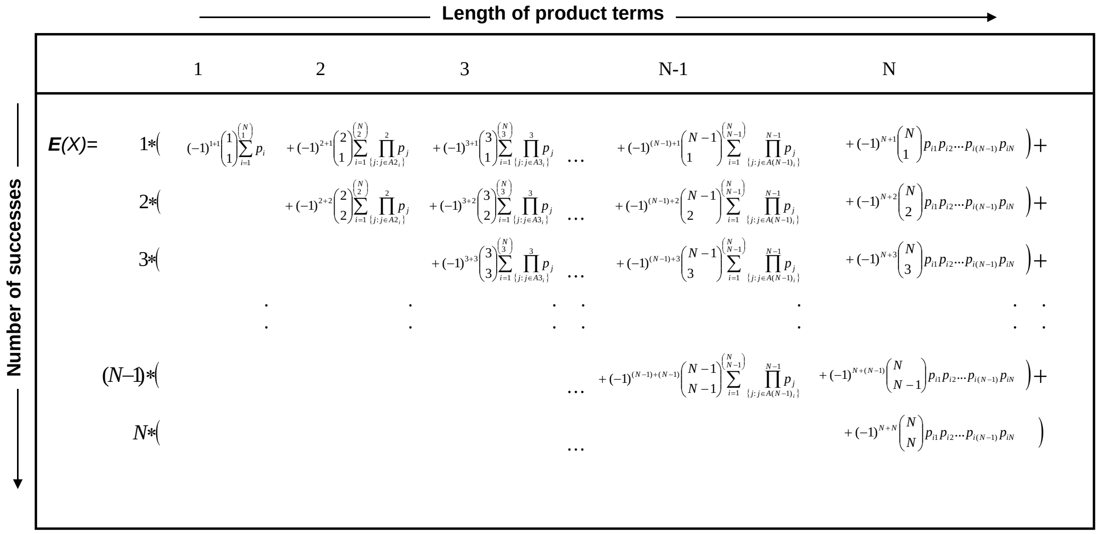

Because of

$$\sum_{k=0}^{n}(-1)^k k\binom{n}{k} = 0 \quad (3)$$

all terms in the same column cancel out except for the term in column 1 which is equal to (2). If co-occurrence probabilities are the same for all lists, (2) becomes:

$$\mu = N * p \quad (4)$$

as expected for the Binomial Distribution.

Similarly, the variance is calculated by multiplying the squared deviation of successes from the mean (#successes - μ)^2 by their respective probabilities and summing over all successes from 0 to N. This calculation evaluates to:

$$\sigma^2 = \sum_{i=1}^{N} p_i - \sum_{i=1}^{N} p_i^2 \quad (5)$$

The proof of (5) is performed exactly as the proof of (2). Thus, the variance is equal to the mean minus the sum of squared co-occurrence probabilities.

For all p_i being equal, we obtain:

$$\sigma^2 = N * p - N * p^2 \quad (6),$$

which is the variance of the Binomial Distribution.
