# Supplementary material for: Identification and Analysis of Co-Occurrence Networks with NetCutter
Source: PLoS One. 2008 Sep 10;3(9):e3178. doi: 10.1371/journal.pone.0003178 (PMC2526157; doi:10.1371/journal.pone.0003178)
Supplement: Simulation S1 — (0.42 MB PDF) [file pone.0003178.s004.pdf]

A simulation study

In order to evaluate the efficacy of NetCutter in uniting genes regulated by common pathways in the same co-occurrence network community when samples of varying size are drawn, a simulation study was conducted. The simulated data set is used to reveal the impact of different user defined parameters onto the overall performance of NetCutter and to address problems connected with multiple testing. Furthermore, the relationship between Poisson-binomial P-values and Z-scores as well as the precision of the bi-binomial approximation used by NetCutter to calculate P-values is discussed.

Generation of simulated data sets

The simulated data sets were generated to model the situation that is commonly observed during meta-analysis of gene expression data, which can be expressed as a set of assumptions. Namely:

1. For each pathway, there are few genes that are strongly regulated and many others that respond with weak deviations from the baseline expression level when the regulating pathway is activated.
2. Different pathways differ by the number of strongly regulated targets.
3. A gene can be regulated by more than one pathway with one pathway providing the domineering regulatory input.
4. A microarray study identifies preferentially the strongly regulated pathway targets and may miss many of the weakly regulated targets. The set of regulated genes identified in a microarray study is called a signature.
5. Different microarray studies produce signatures of varying size.

During meta-analysis, the task is to identify common regulatory inputs for sets of genes from signatures of varying size based on the assumption that genes with common regulatory input will co-occur significantly in different signatures.

In the present simulation, 1000 genes are assumed to be regulated by five different pathways. Each pathway is modeled by an exponential distribution that describes the probability of a gene being identified as differentially regulated in a microarray experiment.

$$P(X = x \mid \lambda) = \lambda e^{-\lambda x}$$

Here, λ is the rate parameter and x is the gene number between 0 and 999 (counting starts at x = 0 for the first gene). This distribution satisfies the first of the above assumptions, i.e. that some genes are strongly regulated while most genes vary weakly upon pathway activation.

The second assumption (different pathways have different numbers of strongly regulated targets) can be accommodated by varying the rate parameter λ for different pathways. Here, λ assumes the values λ_1 = 0.01, λ_2 = 0.009, λ_3 = 0.008, λ_4 = 0.007, and λ_5 = 0.006.

The third assumption (genes are regulated by more than one pathway) is incorporated by changing the most strongly regulated target gene form gene 1 (offset x = 0) for λ_1, to gene 201 (offset x = 200) for λ_2, to gene 401 (offset x = 400) for λ_3, to gene 601 (offset x = 600) for λ_4, to gene 801 (offset x = 800) for λ_5. In other words, P is calculated as λ * exp(-λ*(-offset + x)) for all x >= offset. For the genes preceding this offset (x < offset), P is calculated as λ * exp(-λ*(1000 – offset + x)). The result of this calculation is shown in Fig. 1. As can be read from this Figure, the genes 1 to 200 are mainly regulated by pathways 1 and 5, the genes 201 to 400 are mainly regulated by pathways 2 and 1, the genes 401 to 600 are mainly regulated by pathways 3 and 2, the genes 601 to 800 are mainly regulated by pathways 4 and 3, and the genes 801 to 1000 are mainly regulated by pathways 5 and 4. Each gene receives inputs from all pathways with one pathway domineering and the others having influence at quickly decreasing levels. We expect that genes with similar regulatory input form separate communities in the co-occurrence network. Correctness of community formation can be directly read from the genes names

(e.g. genes 1 to 200 should form a separate community and genes 201 to 1000 should be absent from this community).

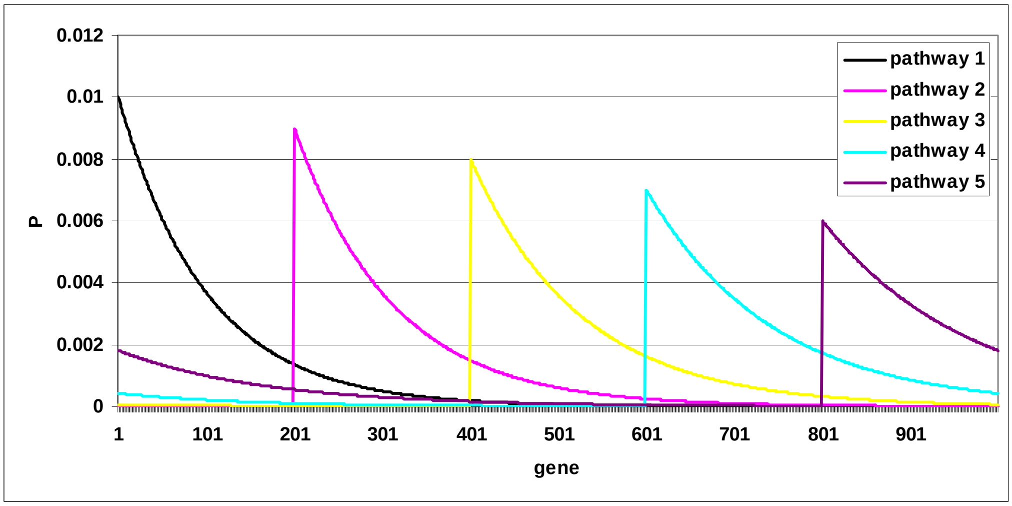

Fig. 1 Regulatory input for each gene by pathways 1 to 5. See text for details.

The assumptions 4 and 5 are incorporated in this model by random sampling from this distribution separately for each pathway. Genes that are most strongly regulated by a pathway will be most likely found in a signature. Finally, samples are of different size varying uniformly between 5 and 100 genes. It should be stressed that this model is just a computational vehicle to test the performance of NetCutter and does not pretend to reflect biological reality in any way.

We generated two different data sets from this model. In the first data set, each pathway was sampled 10 times (equivalent to 10 signatures regarding this pathway, 50 signatures in total, called "scarce data set") and in the second data set each pathway was sampled 50 times (250 signatures in total, called "abundant data set"). The first data set models the situation when data are scarce while the second data set reflects abundance of data. The number of occurrences for each gene in the two data sets is shown in Fig. 2 and 3. Each data set is stored as a set of list (=signature)–entry (=gene) pairs representing a bipartite graph. Both data sets are available at http://bio.ifom-ieo-campus.it/NetCutter/.

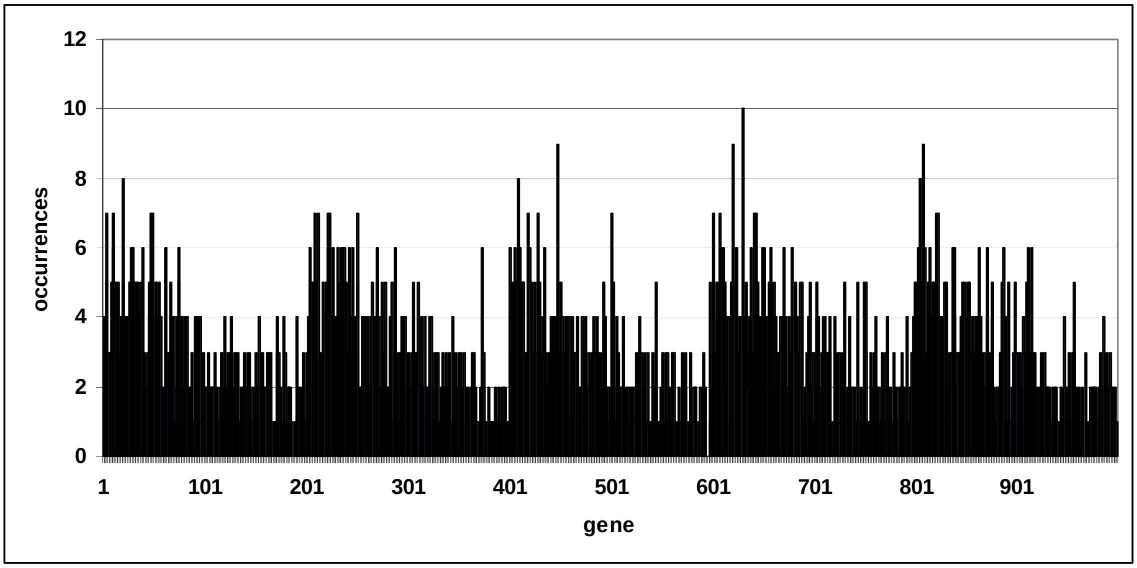

Fig. 2 Number of signatures each gene is found in among 50 signatures. See text for details.

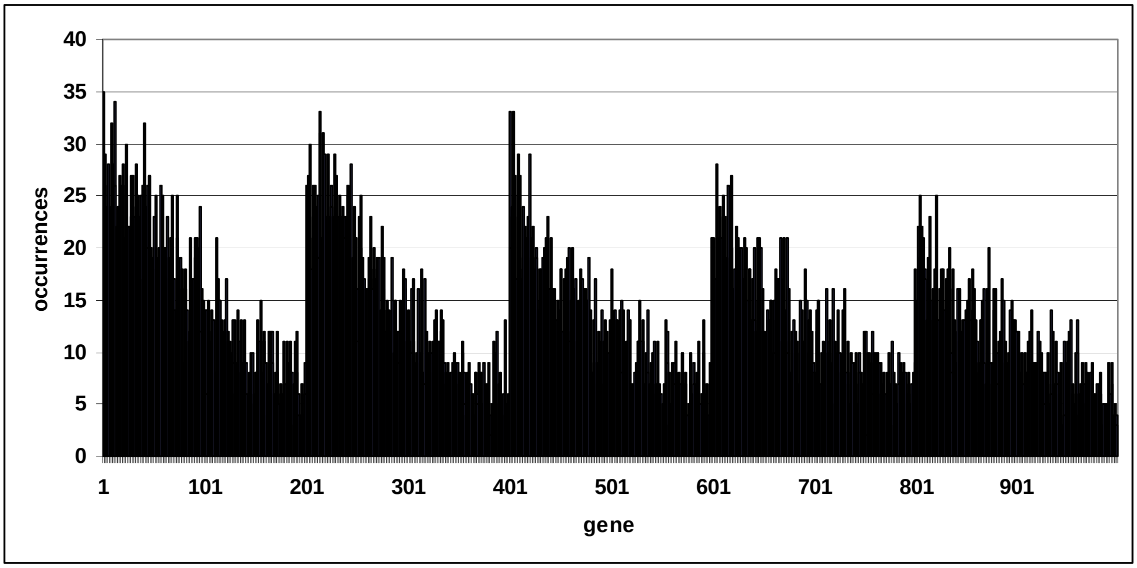

Fig. 3 Number of signatures each gene is found in among 250 signatures. See text for details.

Analysis of simulated data sets

Analysis of a data set starts by loading the bipartite graph into NetCutter followed by determining the occurrence probabilities using the edge-swapping model. For both data sets, 1000 randomized bipartite graphs were analyzed and the number of occurrences of each gene in each list was determined. This number divided by 1000 yields an estimate of the occurrence probability for each gene in each list.

The next step is to determine whether the data set contains useful information. There are three user-defined parameters that can be adjusted such that the number of co-occurrence modules in the real bipartite graph is maximized while the number of co-occurrence modules in a randomized bipartite graph is minimized. The ratio of these numbers provides a signal-to-noise ratio (SNR). When this ratio is significantly greater than 1 for some combination of parameters, the data set likely contains useful information. The three parameters that can be adjusted are the module size, the support, and the significance level. The module size indicates the number of genes that are required to co-occur in the same signature. This number can vary between 2 (pair-wise co-occurrence) and 10. The support parameter indicates in how many lists a combination of genes must be present in as a minimum for being considered further. This number can vary from 1 to the total number of lists. The significance level is determined by setting cut-off values for the Z-score/P-value. NetCutter has a graphical interface plotting the SNRs for any given set of parameter combinations in order to help determining the parameter combination that optimizes the SNR.

Choosing the support level

The support parameter is used to keep the combinatorial explosion associated with analyzing larger module sizes to an acceptable level. However, choosing the support parameter too high is associated with loss of information (see below). Thus, choosing the support parameter is an essential step before analysis with larger module sizes is performed. A useful level of the support parameter can be estimated by running the analysis with module size 2 and support 1 on the real bipartite graph and the randomized version of it. The resulting data can be loaded into NetCutter and higher cut-off values for

the support can be tested for the associated SNR. An example of this procedure for the scarce data set is shown in Table 1.

Table 1 Determining the support parameter. BPG = bipartite graph, SNR = signal-to-noise ratio. Module size = 2, Z = 0 (no cut-off determined)

support	modules in real BPG	modules in randomized BPG	SNR
1	67410	71590	0.94
2	12918	7581	1.70
3	2240	495	4.53
4	312	28	11.14
5	32	2	16.00
6	1	0	1.00

From Table 1 we see that the SNR is significantly larger than 1 at support 3, 4, and 5. Support 6 is clearly too high because only one module is left in the real bipartite graph. At support 2 we will analyze many modules with little gain in information. At support 3 the SNR starts to assume promising levels. Since the SNR will be increased further by analyzing larger module sizes and by setting significance cut-offs accordingly, we choose support 3 for further analysis.

Choosing the significance level

The next question that arises regards the significance cut-off. Since in each NetCutter analysis a real bipartite graph is analyzed in conjunction with a randomized version of this graph, the significance cut-off can be determined as the value that maximizes the SNR. For the scarce data set at support level 3 and module size 2, the SNRs are shown in Fig. 4. We see that at Z = 4 the SNR reaches its maximum of about 10. This means that 1 out of 10 modules would overcome the cut-off by chance alone and thus corresponds to an effective P-value of about 0.1. The effective P-value can be estimated also by examining the P-value cut-off for a single co-occurrence module. The Z-score cut-off of 4 corresponds to a P-value cut-off of 0.9992 for a single module. In total, 117855 modules are being tested at support level 3 (this number can be determined by NetCutter when running the analysis) and we would expect (1-0.9992) * 117855 = 94 modules to pass this cut-off by chance. As shown in Table 2, in the randomized graph we effectively find 41 modules (compared to 440 in the real graph). Thus, NetCutter offers two ways to address the multiple testing problem: comparing the SNR in a real and a randomized

bipartite graph and the calculation of the number of background modules based on P-value cut-offs for single modules and the number of modules being tested. However, in practice, the first approach is easier because it can be addressed graphically without performing calculations. In summary, for module size 2 we choose to run the analysis at support level 3 and Z-score cut-off 4.

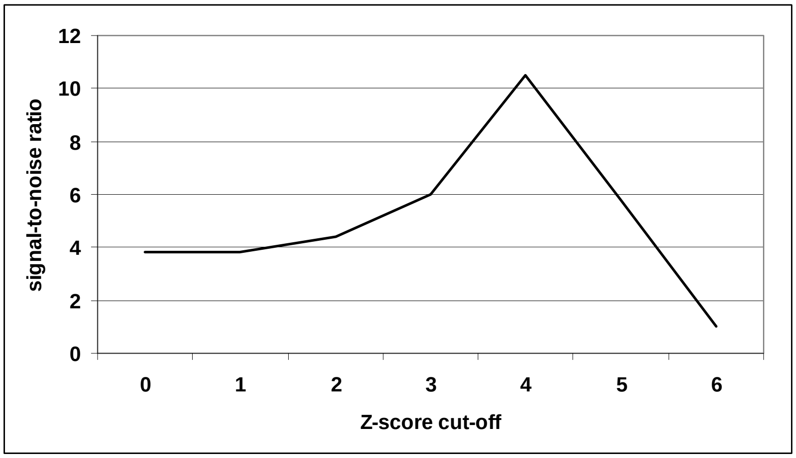

Fig. 4 Signal-to-noise ratio as a function of Z-score cutoff in the scarce data set. Module size 2, support level 3.

<u>*The impact of module size on classification accuracy*</u>

The next step is to run the analysis at support 3 for module sizes 3 to 10. A real and a randomized bipartite graph are analyzed for each module size. The resulting data are loaded into NetCutter and the significance cut-off is adjusted such that the SNR reaches its optimum. From the significant co-occurrence modules, a co-occurrence network is then generated and analyzed for the presence of network communities. In this example, edge-betweenness clustering [1] and eigenvector based clustering [2] are used for this purpose. The data obtained for the scarce data set are summarized in Table 2.

Table 2 illustrates that the SNR grows dramatically with increasing module size up to module size 7. At module sizes larger than 7 signal is lost because the analysis becomes too stringent and the SNR decreases accordingly. At module size 5 and larger, the significance cut-off that was used to adjust the SNR at module size 4 eliminates all

modules in the randomized bipartite graph. Therefore, no further increase of the significance cut-off is necessary because it would only result in loss of signal.

Table 2 Summary of scarce data set analysis: M – module size, Z – Z – score cutoff, P – confidence cutoff, BPG – bipartite graph, SNR - signal-to-noise ratio, EV – eigenvector clustering, EB - edge-betweenness clustering

M	Z	P	modules in real BPG	modules in randomized BPG	SNR	#clusters EV	#clusters EB	edges removed
2	4	0.999	440	41	10.7	47	50	25
3	11	0.999999	1260	10	126	7	6	19
4	20	0.999999999	3443	2	1721.5	5	5	36
5	20	0.999999999	10314	0	10314	5	5	48
6	50	0.999999999	12568	0	12568	6	5	30
7	50	0.999999999	12712	0	12712	6	5	25
8	50	0.999999999	10511	0	10511	4	4	7
9	50	0.999999999	7019	0	7019	3	3	0
10	50	0.999999999	3732	0	3732	3	3	0

The resulting co-occurrence networks have been subjected to community identification analysis using eigenvectors (EV) of the modularity matrix [2] and edge-betweenness (EB) clustering [1]. The number of clusters identified using each approach is listed in Table 2. For EB-clustering, the number of removed edges is also given. The clusters obtained for module size 2 and 3 by EB-clustering are shown in Fig. 5 and 6. It can be seen that at module size 2, the number of clusters identified is roughly 10 times the expected number of clusters (5). This is because many isolated pairs or triples of genes are identified as separate clusters, which cannot be linked to other genes because of lacking edges. The situation improves dramatically for module size 3 (Fig. 6). Here, implicit information is used to link genes that have never co-occurred explicitly. The number of identified clusters is 6 rather than 5 because one isolated cluster composed of 3 genes remains. For module sizes 4 and 5, both EV and EB clustering identify the correct number of clusters. For module sizes 6 and 7, EV clustering breaks up one cluster resulting in 6 identified clusters while EB-clustering still identifies the correct number of clusters. For module sizes 8, 9, and 10 clusters are disappearing because the analysis becomes too stringent.

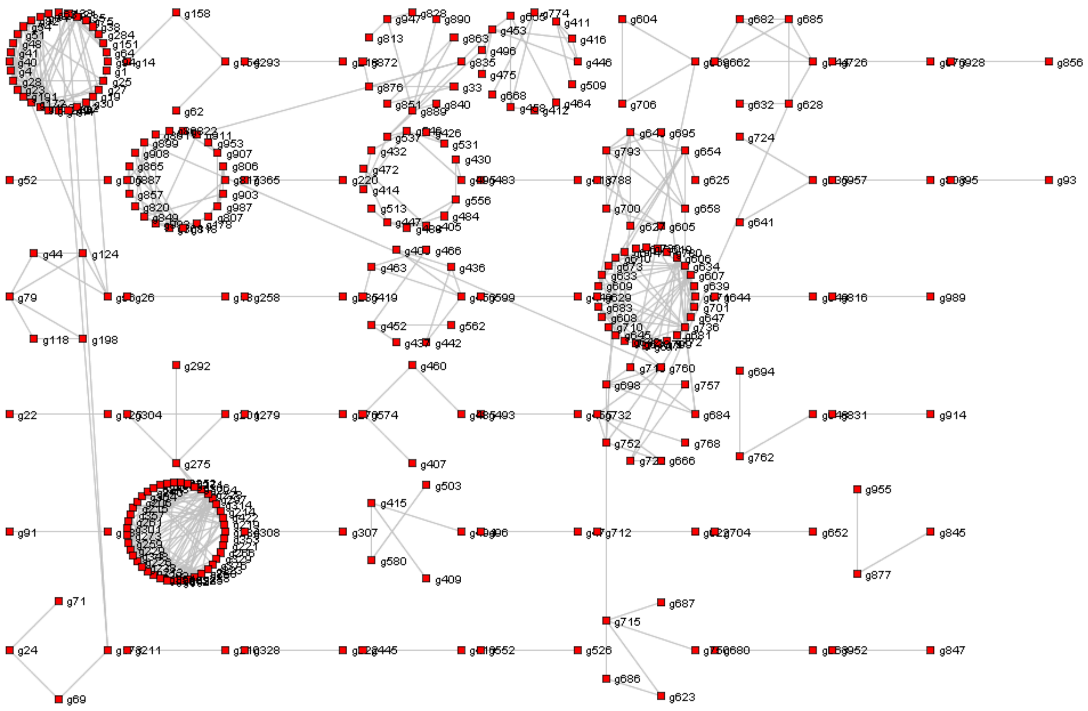

Fig. 5 Edge-betweenness clustering by removing 25 edges from the co-occurrence network of module size 2 in the scarce data set. Underlying pathway structure cannot be identified.

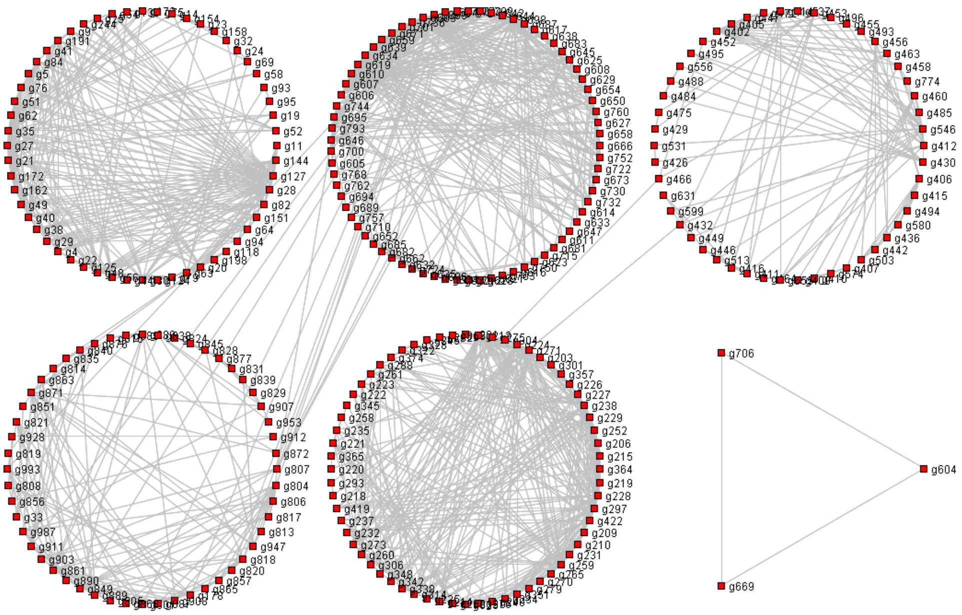

Fig. 6 Edge-betweenness clustering by removing 19 edges from the co-occurrence network of module size 3 in the scarce data set. Underlying pathway structure is clearly visible.

Next, the accuracy of clustering was examined. By design of this data set, genes from 1 to 200 should be linked in the same community and similarly for genes 201 – 400, 401-600, 601-800, and 801-1000.

The accuracy was estimated as follows: For each group of pathway targets the number of genes available for classification was determined. These are the genes that have been found part of significant co-occurrence modules. The genes available for classification can belong to different clusters. The largest cluster in each group of pathway targets was taken as the correct cluster. Genes that were part of any other cluster were taken as false negatives (FN), even when the entire cluster was composed of target genes of the same pathway. Furthermore, genes in the largest (correct) cluster that are targets of a different pathway as the majority of genes in this cluster were taken as false positives (FP). Thus, there are four classes of genes: not classified, classified correctly, false positives, and false negatives. False positives can be understood as “wrong genes in the right cluster” and false negatives as “right genes in the wrong cluster”. The four classes of genes were determined for every group of pathway targets. The results for all five pathways were added and are displayed in Fig. 7 and 8. Fig. 7 shows the results for EB-clustering and Fig. 8 shows the results for EV-clustering.

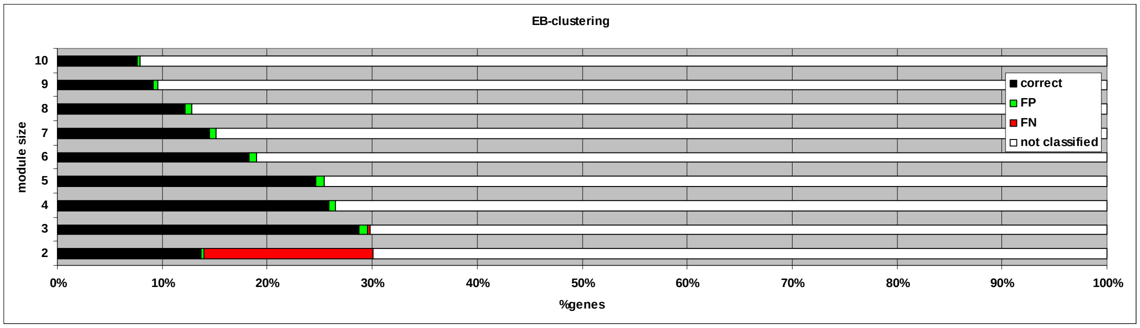

Fig. 7 Accuracy of EB-clustering (edge-betweenness clustering) for scarce data set. FP-false positive, FN-false negative.

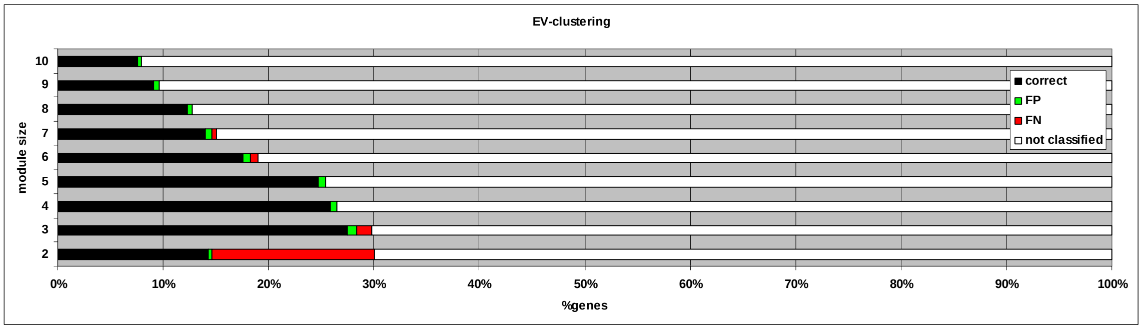

Fig. 8 Accuracy of EV-clustering (eigenvector clustering) for scarce data set. FP-false positive, FN-false negative.

The data in Figure 7 and 8 illustrate that the accuracy of clustering increases sharply with module size 3 without increasing the number of genes that cannot be classified significantly. Considering also the results from Fig. 5 and 6, it is apparent that the large number of misclassified genes with module size 2 is mainly due to many FN errors. These errors can be eliminated by using implicit information obtained at higher module sizes. Both types of clustering perform similarly well, with EB-clustering being slightly more accurate but also much slower. EV-clustering tends to split smaller clusters into sub clusters, thus producing unnecessary FN errors (e.g. module size 6 and 7).

While FN errors can be reduced efficiently by increasing module size, FP errors are largely insensitive to module size. There are at least two possible explanations for the origin of these errors. Either the clustering algorithms do not perform well or the genes that are misclassified as false positives are under-sampled such that there isn't sufficient information to classify them correctly. In the following, we will consider these hypotheses in turn.

Regarding the first hypothesis, we need to evaluate the way the co-occurrence network is constructed. For module sizes larger than 2 the co-occurrence network is built by drawing an edge between each pair-wise combination of genes in a co-occurrence module. This procedure leads to an ordinary undirected graph. EB and EV clustering have been developed to find communities in such graphs. However, one might wonder whether creating an ordinary undirected graph from co-occurrence modules containing more than

two genes is the correct approach. Indeed, one might consider each module a hyperedge and could therefore create a hypergraph by combining all co-occurrence modules. While an ordinary edge is a pair of vertices, a hyperedge is a combination of more than two vertices and a hypergraph is a graph containing hyperedges. Algorithms have been developed to partition hypergraphs. A popular representative of such algorithms is hmetis (http://glaros.dtc.umn.edu/gkhome/metis/hmetis/overview). Thus, we compared the efficacy of EB-clustering to the efficacy of hmetis downloaded from http://glaros.dtc.umn.edu/gkhome/metis/hmetis/download.

The parameters used during hmetis analysis are shown in Table 3. hmetis is designed to partition hypergraphs into partitions of roughly equal size and the number of partitions is a user provided parameter. To allow for imbalances in the size of the partitions, an unbalance (UB) factor must be provided by the user. Choosing this parameter is critical for the performance of hmetis. Therefore, UB factors between 5 and 15 were tested and the results for the UB factors providing the best performance of hmetis are reported. It was found that UB factors lower than 7 and larger than 13 led to strong increases in the error rates while UB factors between 7 and 13 yielded identical results. Only the results for hypergraphs of rank 3 and 4 (corresponding to module size 3 and 4) are reported. The reason is that at higher module sizes the imbalance in the partitions becomes problematic and error rates increase sharply (not shown).

Table 3 Parameters used for hmetis analysis

HGraphFile	filename
Nparts	5 (#clusters)
UBfactor	5 to 15
Nruns	10
CType	1
RType	1
Vcycle	3
Reconst	0
dbglvl	24

The results of the comparison of EB clustering and hypergraph clustering are shown in Table 4. Since hmetis forces all genes into one out of five partitions, false negative results cannot be observed. Therefore, only the total number of misclassified genes for hmetis

and EB-clustering is reported. As can be seen from Table 4, the error rates of both clustering approaches are similar. At module size 3, hmetis classifies one more gene correctly as compared to EB. However, 11 genes remain misclassified as compared to 12 genes for EB-clustering. At module size 4, hmetis makes 10 errors as compared to 6 errors by EB-clustering. Interestingly, however, the misclassified genes are strongly overlapping. This observation argues in favor of the hypothesis that these false positive errors are mainly caused by sampling effects that make the co-occurrence pattern of misclassified genes similar to the corresponding pattern of the genes in the cluster they are found in.

Table 4 Hypergraph (hmetis)- and EB-clustering for module size (M) 3 and 4. Misclassified genes are shown

	hmetis M3	EB M3	hmetis M4	EB M4
1	33	33		
2	178	178	178	
3			230	
4	244	244	244	244
5			251	
6	419	419	419	419
7	422	422	422	422
8	604	604		
9	631	631		
10	655	655	655	655
11	669	669		
12	706	706		
13	774	774	774	774
14			807	
15		899	899	899

To investigate the second hypothesis, i.e. that better sampling reduces false positive errors, we used the data set where each pathway was sampled 50 instead of 10 times (the abundant data set). The analysis was performed exactly as described for the scarce data set. The results are shown in Fig. 9 and 10.

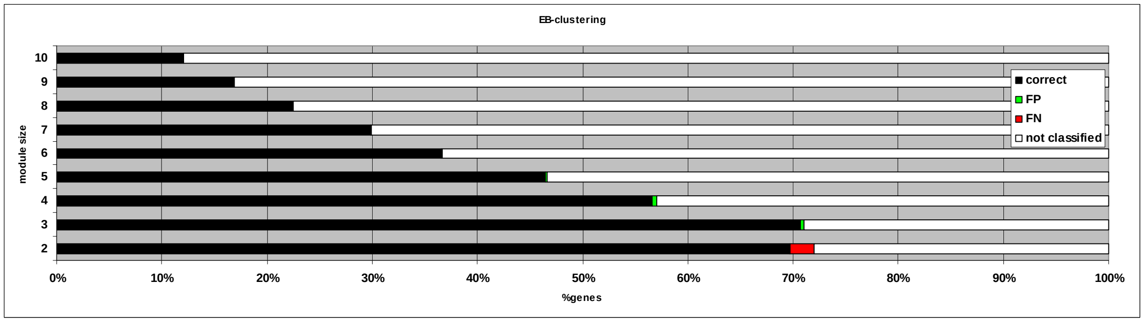

Fig.9 EB clustering of the abundant data set. FP-false positives, FN-false negatives

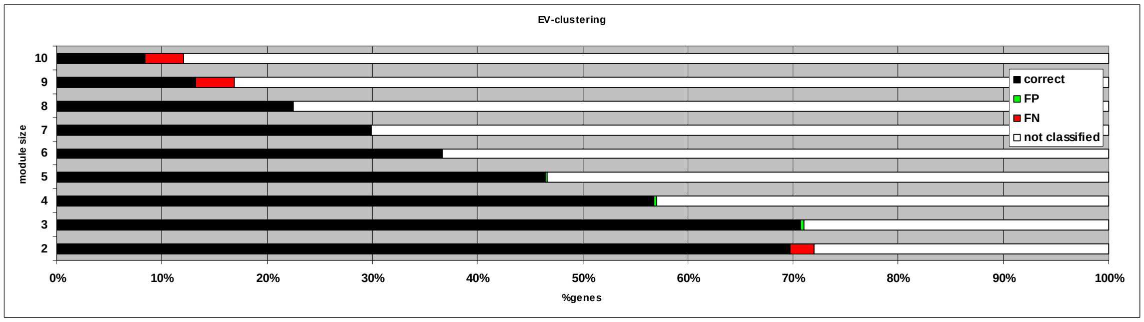

Fig. 10 EV-clustering of the abundant data set. FP-false positives, FN-false negatives

Comparing Fig. 7 and 8 to Fig. 9 and 10, it can be seen that false negative errors, that were very abundant at module size 2 in the scarce data set, are strongly reduced by deeper sampling of the pathways. Furthermore, false positive errors have virtually disappeared. Only two to four genes out of more than 700 are misclassified using module size 3. At module sizes larger than 4, false positive errors are completely absent. There are some false negative errors at module size 9 and 10 by EV-clustering, which is due to the tendency of EV-clustering to split up poorly interconnected communities. In practice, module sizes 9 and 10 are rarely used, however, because of their excessive stringency. Moreover, the number of genes that are available for classification increases from 300 to more than 700. The number of identified clusters in the abundant data set is shown in Table 5. Due to the reduced rate of false negatives at module size 2 in the abundant data set, 6 and 15 clusters are being identified by EV- and EB-clustering, respectively, compared to 47 and 50 in the scarce data set. At module size 3 and 4, the correct number of 5 clusters is being identified with marginal rates of FP errors. At higher module sizes, clusters can be split or disappear, as in the scarce data set.

Table 5 Number of clusters in the abundant data set as a function of module size

module size	# EV clusters	# EB clusters
M2	6	15
M3	5	5
M4	5	5
M5	5	6
M6	4	4
M7	4	4
M8	3	3
M9	4	3
M10	3	2

Impact of analysis stringency on cluster size and number

The next aspect to be discussed in this simulation study is the impact of analysis stringency on cluster size and cluster number. As was briefly mentioned above, the support parameter can be used to limit computational complexity associated with analyses using large module sizes. However, when the support parameter is chosen too high, significant loss of information can result. This is shown in Fig. 11. Here, the analysis was performed on the abundant data set at module size 3, Z = 5, P = 0.9999, and the support parameter was varied from 5 to 10. It can be seen that the cluster sizes get increasingly smaller and clusters start to disappear completely at support 9 and 10. The first clusters to disappear are those with smaller rate parameter λ in the exponential distribution. A smaller rate parameter means that the exponential distribution decays more slowly and that the pathway regulates more target genes with similar strength. As a consequence, during the sampling process more genes have a similar opportunity to be present in the signature and a specific gene will co-occur less frequently with other genes, leading to more insignificant co-occurrence modules and a smaller cluster for that pathway. The same argument applies to increases in stringency due to larger module sizes. In practice, the number of clusters actually present in the data should be estimated by running the analysis at different levels of module size and support. As a rule of thumb, module sizes between 3 and 5 should give the correct answer when they reveal the same number of clusters at different levels of support. In any case, analysis at module size 2 is very likely to over-estimate the number of clusters and analysis at module sizes larger than 6 will miss clusters with low rate parameters.

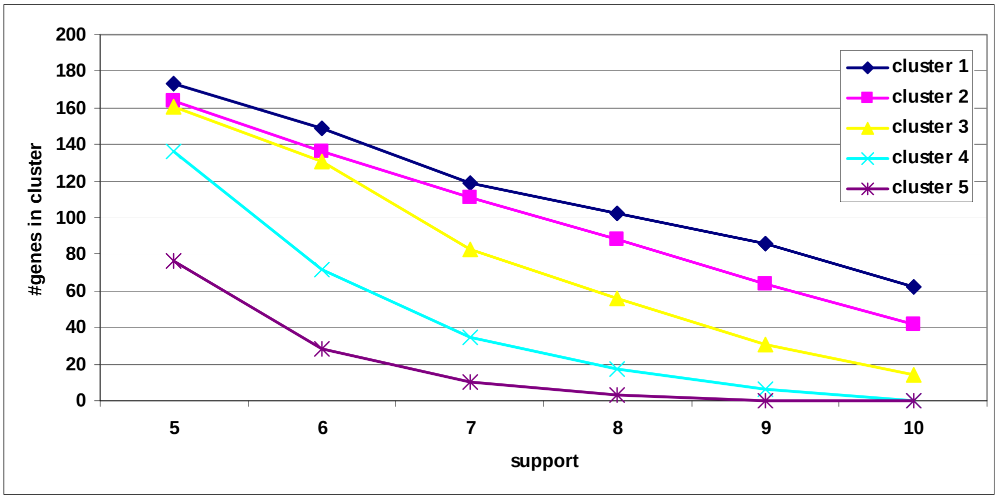

Fig. 11 Cluster sizes as a function of support parameter. Module size 3, Z = 5, P = 0,9999.

Precision of the Bi-binomial approximation

The user of NetCutter might be confused by the use of Z-scores and P-values to adjust the significance cut-off. What is the relationship between them? The bi-binomial approximation has been developed because Z-scores that are not normally distributed, such as binomial and Poisson-binomial Z-scores used here, do not correspond to the same P-values for different probabilities of success. Since P-values permit precise determination of confidence intervals, they are preferable to Z-scores. However, Poisson-binomial P-values are difficult to calculate exactly and therefore an approximation is needed. The relationship between Z-scores and P-values for Poisson-binomial distributions corresponding to sets of Poisson trials of equal size and mean but with increasing variability in the probabilities of success from trial to trial is shown in Fig. 12. It can be seen that the cumulative distribution function is shifting to the left as the variability of success probabilities increases. As a consequence, Z-scores under-estimate the significance of large numbers of success and over-estimate the significance of small numbers of success as compared to the corresponding binomial distribution (standard error 0.5).

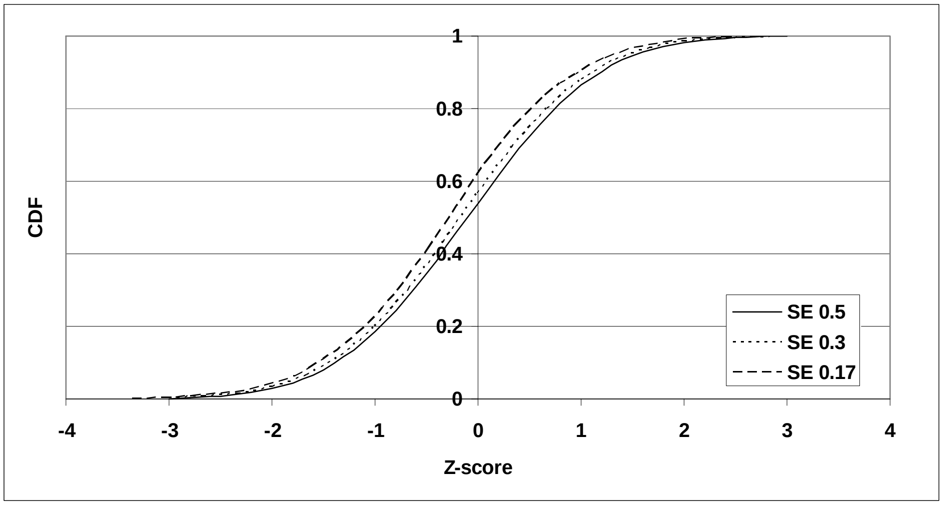

Fig. 12 Relationship between Poisson-binomial Z-scores and P-values. The cumulative distribution function (CDF) of increasingly narrow Poisson-binomial distributions with equal mean is shown as a function of corresponding Z-scores. SE – standard error.

This effect becomes less important for large absolute values of Z-scores. In practical terms, the use of Z-scores or P-values to adjust the SNR in a NetCutter analysis are largely equivalent. However, when the strength of associations between communities of genes and gene lists is studied, exact levels of confidence are needed. Here, P-values are preferred. Since NetCutter calculates bi-binomial P-values, the question about the precision of the bi-binomial approximation of Poisson-binomial P-values arises.

We studied the precision of the bi-binomial approximation for symmetric and asymmetric Poisson-binomial distributions for different amounts of variability in the probabilities of success and different numbers of Poisson trials. Exact Poisson-binomial P-values were calculated using the procedures reported by [3]. The cumulative distribution functions were calculated for the Poisson-binomial and the bi-binomial distributions and the difference between them was plotted as a function of P-value in each case.

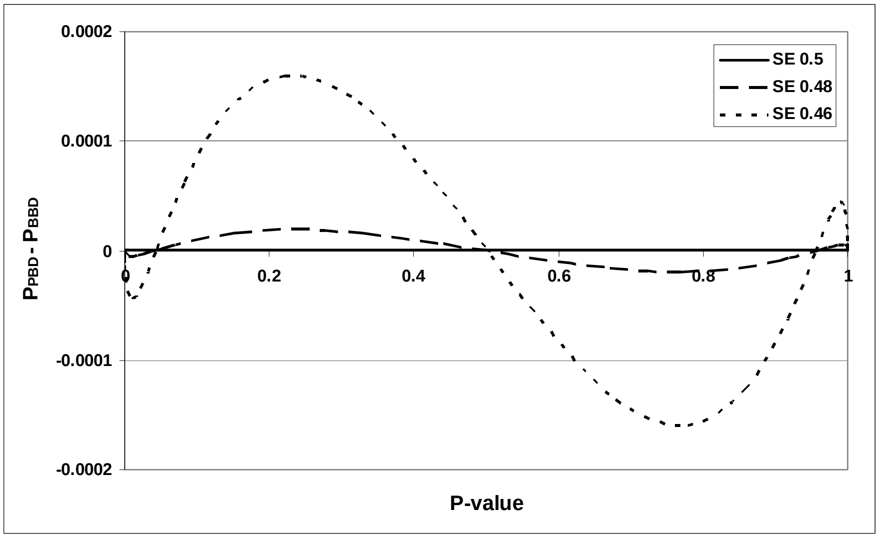

Fig. 13 Precision of bi-binomial distribution (BBD) for increasingly narrow symmetric Poisson-binomial distributions (PBD). Number of Poisson trials is 50. Mean is 25. SE – standard error.

Fig. 13 shows that BBD exactly reproduces the binomial distribution (SE = 0.5, curve coincides with X-axis) but imprecision is observed for increasing variability in the probabilities of success, measured here as decreasing standard error (square root of Poisson-binomial variance divided by number of trials). The imprecision is largest for insignificant P-values and quickly vanishes as P-values approach 0 or 1. For commonly used confidence levels of 0.95 or 0.99, the BBD is precise to 4 digits after the comma.

How is the precision influenced by the number of trials? We studied this question for symmetric Poisson-binomial distributions with standard error of 0.48 for 50, 100, and 150 trials. The results are shown in Fig. 14 and show that the precision increases with the number of trials. BBD is therefore complementary to the procedures reported by [3], which suffer from numeric instability for large numbers of trials.

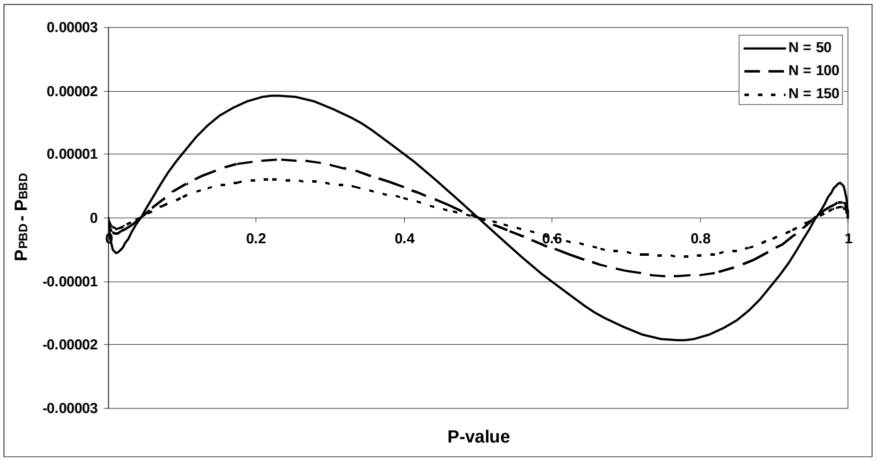

Fig. 13 Precision of bi-binomial distribution (BBD) for symmetric Poisson-binomial distributions (PBD) with increasing number of trials. Standard error – 0.48.

During meta-analysis of gene expression data represented as bipartite graphs, the bipartite graphs are generally sparse because a specific gene is generally found differentially regulated in only a few studies. As a consequence, occurrence probabilities of genes per list are much smaller than 0.5, which leads to asymmetric Poisson-binomial distributions. We studied the precision of BBD for a set of Poisson trials with average probability of success equal to 0.2 for 20, 40, and 80 trials and standard error of 0.4. The results are shown in Fig. 14.

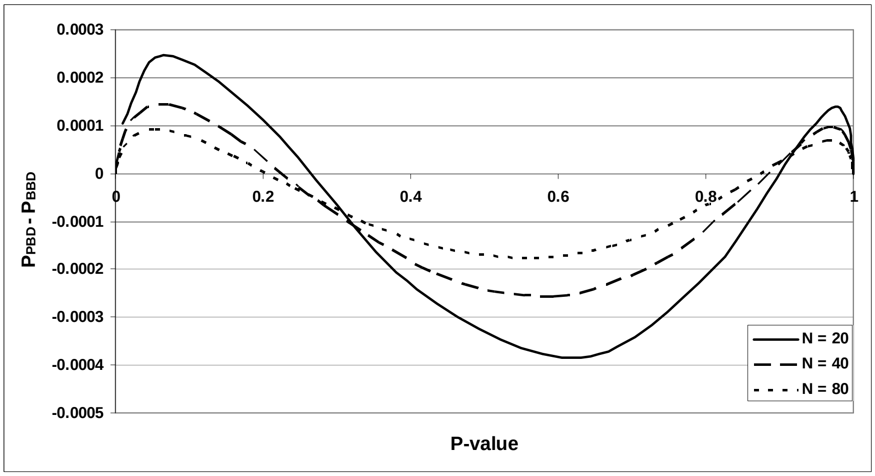

Fig. 14 Precision of bi-binomial distribution (BBD) for asymmetric Poisson-binomial distributions (PBD) with increasing number of trials. Standard error – 0.4.

As can be seen from Fig. 14, the imprecision of BBD is smaller for large P-values than for small P-values when the average of the trial probabilities is smaller than 0.5 (vice versa if the average of the trial probabilities is larger than 0.5, not shown).

The results shown in Fig. 12 to Fig. 14 indicate that the precision of BBD has a number of properties that make BBD a useful tool for the analysis of gene expression data: The imprecision is largely confined to insignificant P-values, the precision grows for large numbers of trials, and the precision for asymmetric Poisson-binomial distributions with average trial probabilities smaller than 0.5 are more pronounced for small P-values rather than for large P-values, which are used by NetCutter to identify significant co-occurrence modules. Furthermore, the imprecision of BBD disappears for large P-values. Indeed, the imprecision of BBD for a gene in the PubLiME data set [4] composed of 231 lists, average occurrence probability of 0.04, and standard error of 0.18 is found to be 1E-5 for P-values of 0.998 and much smaller that that for larger P-values.

Conclusions

The main conclusion from this simulation study is that co-occurrence analysis at module sizes larger than two is much more effective in identifying genes regulated by common pathways than pair-wise co-occurrence analysis. The main reason is that at module sizes larger than two implicit relationships between co-occurring genes can be exploited to classify genes. Module size 3 appears to offer the best compromise between accuracy of classification and loss of classifiable genes due to increased stringency of analysis. The use of implicit relationships leads to a dramatic reduction in the rate of false negative classification errors.

False positive errors, on the other hand, are mainly caused by under-sampling of the data set and cannot be eliminated by increases in module size or by using hypergraph clustering approaches. While hypergraph clustering may be advantageous in some circumstances, it is also more computationally demanding. During hypergraph clustering, each significant co-occurrence module represents a separate hyperedge. Since the number of significant co-occurrence modules in a typical analysis can be hundreds of thousands,

the representation of the co-occurrence data as a conventional undirected graph by drawing an edge between each pair-wise combination of genes in a co-occurrence module leads to a significant reduction of the number of edges without detectable loss of classification accuracy. Indeed, NetCutter identifies the correct number of clusters in the scarce data set with surprisingly few false positive errors.

This simulation illustrates the impact of analysis stringency on the number and the size of clusters identifiable in a data set. Perhaps somewhat counter-intuitively, pathways that regulate more genes with similar strength are more difficult to identify by co-occurrence analysis, particularly when data are scarce.

Finally, we have shown that the precision of the bi-binomial approximation of Poisson-binomial P-values allows reliable determination confidence levels. The precision observed for genes in the PubLiME data set is in the order of 1E-5. In practice, the user should rely on BBD P-values when the strength of associations between network communities and gene lists is analyzed because exact confidence levels cannot be derived from Z-scores alone. For the purpose of adjusting the SNR in a NetCutter analysis, Z-scores and P-values are equally effective with the difference that Z-scores are much faster to calculate. The user may choose to shut off P-value calculation for large data sets to accelerate co-occurrence analysis.

References

1. Newman ME, Girvan M (2004) Finding and evaluating community structure in networks. Phys Rev E Stat Nonlin Soft Matter Phys 69: 026113.
2. Newman ME (2006) Finding community structure in networks using the eigenvectors of matrices. Phys Rev E Stat Nonlin Soft Matter Phys 74: 036104.
3. Chen SX, Liu JS (1997) Statistical Applications of the Poisson-Binomial and Conditional Bernoulli Distributions. Statistica Sinica 7: 875-892.
4. Finocchiaro G, Mancuso FM, Cittaro D, Muller H (2007) Graph-based identification of cancer signaling pathways from published gene expression signatures using PubLiME. Nucleic Acids Res 35: 2343-2355.
